# BMP and WNT signalling cooperate through LEF1 in the neuronal specification of adult hippocampal neural stem and progenitor cells

**DOI:** 10.1038/s41598-018-27581-0

**Published:** 2018-06-18

**Authors:** Tomás Armenteros, Zoraida Andreu, Rafael Hortigüela, D. Chichung Lie, Helena Mira

**Affiliations:** 1 Stem cells and Aging Unit, Instituto de Biomedicina de Valencia, Consejo Superior de Investigaciones Científicas, València, Spain; 2 Centro de Investigación Príncipe Felipe, València, Spain; 3 Chronic Disease Programme, Instituto de Salud Carlos III, Majadahonda, Spain; 4 Institute of Biochemistry, Emil Fischer Center, Friedrich-Alexander-Universität Erlangen-Nürnberg, Erlangen, Germany

## Abstract

Neuronal production from neural stem cells persists during adulthood in the subgranular zone of the hippocampal dentate gyrus. Extracellular signals provided by the hippocampal microenvironment regulate the neuronal fate commitment of the stem cell progeny. To date, the identity of those signals and their crosstalk has been only partially resolved. Here we show that adult rat hippocampal neural stem and progenitor cells (AH-NSPCs) express receptors for bone morphogenetic proteins (BMPs) and that the BMP/P-Smad pathway is active in AH-NSPCs undergoing differentiation towards the neuronal lineage. *In vitro*, exposure to the BMP2 and BMP4 ligands is sufficient to increase neurogenesis from AH-NSPCs in a WNT dependent manner while decreasing oligodendrogenesis. Moreover, BMP2/4 and WNT3A, a key regulator of adult hippocampal neurogenesis, cooperate to further enhance neuronal production. Our data point to a mechanistic convergence of the BMP and WNT pathways at the level of the T-cell factor/lymphoid enhancer factor gene *Lef1*. Altogether, we provide evidence that BMP signalling is an important regulator for the neuronal fate specification of AH-NSPCs cultures and we show that it significantly cooperates with the previously described master regulator of adult hippocampal neurogenesis, the WNT signalling pathway.

## Introduction

Neurogenesis in mammals persists throughout adult life in specific brain locations or “niches” due to the existence of reservoirs of largely quiescent neural stem cells (NSCs) that can generate new neurons through a series of intermediate progenitors. The two main niches in rodents are located in the subgranular zone (SGZ) of the hippocampal dentate gyrus and in the ventricular-subventricular zone or subependymal zone (SEZ) of the lateral ventricles [reviewed in^1,2^]. The activation of quiescent neural stem cells to give rise to progenitor cells, their expansion, neuronal cell fate determination, migration and full differentiation are the main sequential phases required for proper adult neurogenesis. Local niche signals from the neural stem cell microenvironment dynamically regulate all these phases [reviewed in^3^]. Among these signals, short-range niche morphogens, including ligands from the WNT and Bone morphogenetic protein (BMP)/Growth and Differentiation Factor (GDF) families, are thought to play a prominent modulatory role.

WNT ligands are secreted glycoproteins implicated in a variety of brain developmental processes^4,5^. WNTs signal through the Frizzled receptors and several co-receptors, such as LRP5 and LRP6, and elicit both canonical and non-canonical signalling pathways. In the canonical (β-catenin-dependent) pathway, WNT leads to the stabilization of β-catenin and its entrance into the nucleus where it binds to T-cell factor/lymphoid enhancer-binding factor (TCF/LEF) transcription factors, activating WNT target genes^6^. Adult NSCs and progenitor cells secrete functional ligands that stimulate a baseline WNT autocrine signalling necessary for maintaining multipotency^7,8^. Concomitantly, WNT ligands are also released from niche astroglial cells, exerting paracrine effects on NSCs/progenitors that regulate some of the sequential steps of adult neurogenesis. As an example, WNT7A secreted from SEZ astrocytes promotes NSC self-renewal through the non-canonical signalling pathway^9^ whereas WNT3 secreted from hippocampal astrocytes promotes neuronal differentiation through the canonical signalling pathway^10^. *In vivo* studies also demonstrate that the β-catenin-mediated canonical signalling is required for proper adult neurogenesis^11^. Moreover, WNTs are key for the terminal differentiation of the newborn neurons and control late phases of adult neurogenesis, such as dendritogenesis and migration^12,13^. Despite this prominent role of the WNT pathway in adult neurogenesis, its interaction with other niche signalling pathways remains poorly characterized.

The BMP/GDF signalling pathway plays a crucial role in regulating the adult neurogenesis process^3^. BMPs and GDFs are the largest subfamily of the TGF-β ligand superfamily. Two of the BMP/GDF subgroups, the Dpp class (BMP2/4) and the 60 A class (BMP5-8) markedly influence neurogenesis during brain development, but their precise function in adult neurogenesis remains less explored. BMP ligands signal through a heterotetrameric complex formed by two types of Ser–Thr kinase receptors (type 1 and type 2 receptors). *In vitro* binding assays have shown that type 2 receptors (BMPR2, Act-RIIA, Act-RIIB) interact similarly with all BMP ligands from the Dpp and 60 A class. However, type 1 receptors bind the ligands with variable affinities and consequently, the specificity in ligand recognition is dictated through the identity of the BMP type 1 receptor expressed by the cells. There are three main type 1 receptor family members: BMPR1A (ALK3), with high affinity for the Dpp protein family^14^, and BMPR1B (ALK6) and ACVR1 (ALK2), with affinity for the 60 A protein family^14–16^. Regardless of the combination of type 1/type 2 receptors in the heterotetrameric complex, the ligand-receptor interactions can trigger either the canonical (SMAD-dependent) or the non-canonical (SMAD-independent) signalling pathways^17^. In the canonical pathway, SMAD1, 5 and 8 are phosphorylated at the C-terminus by the activated type 1 receptor and then complex with SMAD4 and translocate into the nucleus. The complex interacts with co-activators or co-repressors to regulate gene expression. In the adult hippocampus, several studies have established a principal role for the type 1 receptor BMPR1A and for canonical BMP signalling in regulating the balance between NSC quiescence and proliferation^18–22^. However, the function of this family of morphogens and receptors in neuronal fate determination during adulthood remains less characterized.

Herein, we investigated the role of canonical BMP signalling in promoting neurogenesis from adult rat hippocampal neural stem and progenitor cells (AH-NSPCs). We show that a short exposure to BMP ligands from the Dpp class (BMP2 and BMP4) elicits the SMAD-dependent canonical signalling pathway in AH-NSPCs, which is sufficient to specify the neuronal fate of the stem cell progeny while decreasing oligodendrogenesis, but without affecting the astrocyte fate. Overexpression of a constitutive active form of the type 1 receptor BMPR1A recapitulates the phenotype. The increase in neurogenesis triggered by BMP2/4 requires endogenous canonical WNT signalling. We also describe in detail a synergistic crosstalk between the BMP and WNT canonical signalling that leads to an increase in neurogenesis, and we provide evidence for a role of the transcription factor LEF1 in the mechanistic convergence of the BMP and WNT pathways.

## Experimental Procedures

### Animals

2 month old Crl:CD1 males were used to dissect the hippocampal dentate gyrus. Mice were maintained under SPF conditions and all manipulations were approved by the Committee for Research Ethics and Animal Welfare of the Instituto de Salud Carlos III, Spain. All experiments were performed in accordance with the Spanish and European guidelines and regulations (RD53/2013).

### Cell Culture

For proliferation and differentiation assays we used rat Adult Hippocampal Neural Stem and Progenitor Cells (AH-NSPC)^23^. AH-NSPCs were maintained in N2 medium, DMEM/F-12(1:1) (Gibco) adding N2 Supplement (100×) (Gibco), with 20 ng/ml of human fibroblast growth factor 2 (FGF-2) (PeproTech), growing in poly-ornitine (10 µg/ml)/laminin (5 µg/ml) (Sigma-Aldrich/Millipore) coated dishes (Hsieh *et al*., 2004). For the differentiation assays AH-NSPCs were cultured in MW24 dishes using poly-ornitine/laminin treated 12 mm cover glasses (ThermoScientific), recombinant BMP2, BMP4, BMP7 and WNT3A (PeproTech) were added at the indicated concentration, using 1 µM Retinoic Acid (Sigma-Aldrich) and 5 µM Forskolin (Sigma-Aldrich) as positive control^23^. In those experiments in which we inhibited the p38 MAPK pathway we used 1 µM of SB203580 (Abcam). To inhibit the β-catenin-dependent WNT canonical pathway we used 1 and 5 µM of XAV939 (Sigma).The caBMPR1a retroviral vector was kindly provided by Kinichi Nakashima, Kyushu University, Japan. The GFP and LEF1-GFP lentiviral vectors were from OriGene.

### Immunostaining

Cultured cells were fixed with 2% paraformaldehyde (Panreac). Samples were incubated with blocking solution (10% Fetal Bovine Serum, 0.2% Triton-X100). Primary and secondary antibodies used for the stainings are as follows: Monoclonal Anti-Neuronal Class III β-Tubulin 1:250 (Covance, Ref. mms-435p), Monoclonal Anti-Glial Fibrillary Acidic Protein 1:300 (Sigma-Aldrich, Ref. g3893), Polyclonal Anti-Glial Fibrillary Acidic Protein 1:300 (Dako, Ref. z0334), Anti-Myelin Basic Protein 1:40 (Abcam, Ref. ab2404), Phospho-Smad1/Smad5/Smad8 Antibody 1:100 (Cell Signalling, Ref. 9511), Monoclonal Anti-O4 1:150 (Sigma-Aldrich, Ref. MAB345), Goat Anti-Doublecortin (C-18) (Santa Cruz Biotechnology, Ref. sc8066), Monoclonal Anti-Nestin [Rat-401] 1:500 (Abcam, Ref. ab11306), Polyclonal Goat Anti-SOX2 1:300 (R&D Systems, Ref. af2018), Rabbit monoclonal anti-Ki67 1:50 (Thermo Scientific, Ref. RM-9106), Monoclonal Anti-MAP2 (2a + 2b) clone AP-20 1:300 (Sigma-Aldrich, Ref. M1406), Monoclonal anti-ID1 (B-8) 1:100 (Santa Cruz Biotechnology, Ref. sc133104), Cy3 Goat anti-Mouse 1:2000 (Jackson ImmunoResearch, Ref. 115-166-062), Cy3 Donkey anti-Rabbit 1:2000 (Jackson ImmunoResearch, Ref. 711-165-152), Alexa 488 Donkey anti-Rabbit 1:500 (Jackson ImmunoResearch, Ref. 711-546-152), Biotinylated Donkey Horse anti-Goat 1:200 (Vector Laboratories, Ref. BA-9500) and Cy5 Streptavidin 1:400 (Jackson ImmunoResearch, Ref. 016-170-084). The RIP monoclonal antibody developed by S. Hockfield was obtained from the Developmental Studies Hybridoma Bank, created by the NICHD of the NIH and maintained at The University of Iowa. DNA was stained with 4,6-diamidino-2-phenylindole (DAPI) 10 µg/ml (Sigma). Samples were processed with Dabco/Mowiol 1/20 (Sigma/Merck), and analysed with an ImageA1 Axio microscope (Carl Zeiss).

### Gene Expression analysis

RNA was extracted from cells or 2-month old wild type (CD-1) mice using Illustra RNAspin Mini Kit (GE Healthcare) and measured with a Microplate Reader (Infinite M200 TECAN). cDNA was obtained by reverse-transcription (RT) employing PrimeScrip RT Reagent kit (Takara). Gene expression was determined by quantitative polymerase chain reaction (qPCR) in a LightCycler480 (Roche) using SYBR PremixEX Taq (2×) (Takara) and the corresponding forward and reverse primer for each gene, following the manufacturer protocol. Data were analysed according to the 2^−ΔΔCt^ method^24^. Primer sequences are available upon request.

### Western-blot assays

Cell extracts were fractionated by SDS-PAGE and transferred to a polyvinylidene difluoride (PVDF) membrane following the manufacturer’s protocol (Bio-Rad). Membranes were incubated with 5% nonfat milk or 3% BSA (Sigma) in TBST for 60 min. Primary and secondary antibodies used for the western-blot assays are as follows: Phospho-Smad1/Smad5/Smad8 antibody (Cell Signalling, Ref. 9511 and Ref. 13820), Smad1/5/8 (N-18)-R antibody (Santa Cruz Biotechnology, Ref. sc-6031-r), Neuronal Class III β-Tubulin antibody (Covance, Ref. mms-435p), Glial Fibrillary Acidic Protein antibody (Dako, Ref. z0334), Goat Doublecortin (C-18) antibody (Santa Cruz Biotechnology, Ref. sc8066), Phospho-LRP6 (Ser1490) antibody (Cell Signalling, Ref. 2568), β-actin antibody (Sigma-Aldrich, Ref. a5441), Donkey anti-Rabbit ECL 1:50000 (Amersham, Ref. na934), Sheep anti-Mouse ECL 1:50000 (Amersham, Ref. na931), Donkey anti-Goat HRP 1:10000 (Santa Cruz Biotechnology, Ref. sc-2020).

### Phylogenetic tree and *Lef1* Promoter Characterization

Phylogenetic Tree distances between BMPs were calculated using CLUSTAL-W2 (http://www.ebi.ac.uk/Tools/msa/clustalw2) using default settings and the alignment viewer *AliView*. The accession numbers of the BMP protein sequences analysed in mouse were as follows: BMP2 (NP_031579.2), BMP4 (AAH13459.1), BMP5 (NP_031581.2), BMP6 (NP_031582.1), BMP7 (NP_031583.2), BMP8A (NP_031584.1) and BMP8B (NP_031585.2). The 5 kb sequence upstream of the initial ATG start codon from the *Rattus norvegicus Lef1* gene was retrieved (giІ389673387:821409826409), and promoter and transcriptional factor binding sites analysis were carried out using *Genomatix Promoter Inspector* and *MatInspector* tool (*Genomatix Software Suite v3*.*0)*.

### Chromatin ImmunoPrecipitation (ChIP)

AH-NSPC were treated with BMP4 (25 ng/ml) for 6 h. Proteins and DNA were crosslinked for 10 mins with formaldehyde 37% (Panreac) to a final concentration of 1%. Extracted material was sonicated using a UP50H Ultrasonic Processor (Hielscher), sonication conditions were as follow: 30% amplitude, 10 sec x20 pulses. Proteins were pulled down using 60 µl Protein A agarose/Salmon Sperm DNA (Millipore) and 10 µg of Smad4 Antibody (H-552) (Santa Cruz Biotechnology, Ref. sc-7154). ChIP Dilution Buffer, Low Salt Immune Complex Wash Buffer, Hight Salt Immune Complex Wash Buffer, LiCl Immune Complex Wash Buffer, TE Buffer and SDS Lysis Buffer were prepared as described in Chromatin Immunoprecipitation (ChIP) Assay Kit (Upstate). Immunoprecipitated material was amplified by PCR using DNA polymerase (Biotools), 10× Standard Reaction Buffer with 2 mM MgCl_2_ (Biotools), 240 µM dNTP Mix (Invitrogen) and *Lef1* promoter primers (sequences available upon request).

### Statistical Analysis

The statistical significance of the difference between means for the kinetics experiments was assessed by one-way ANOVA, using the Tukey test as post-hoc comparison. To determine the significance of the BMP2/4 and WNT3A synergic effect we used two-way ANOVA analysis of the percentage of neurons. The significance between means of the remaining experiments was calculated using paired 2-tailed Student *t* test. All the values correspond to average ± sem, and those with a *P* value < 0.05 were considered significant (^*^P < 0.05; ^**^P < 0.01; ^***^P < 0.001). Arcsen transformation was carried out for the statistical analysis of percentages.

## Results

### BMP ligands and BMP receptors are expressed in the adult hippocampal dentate gyrus and are differentially regulated during AH‐NSPC differentiation

**Figure 1:**
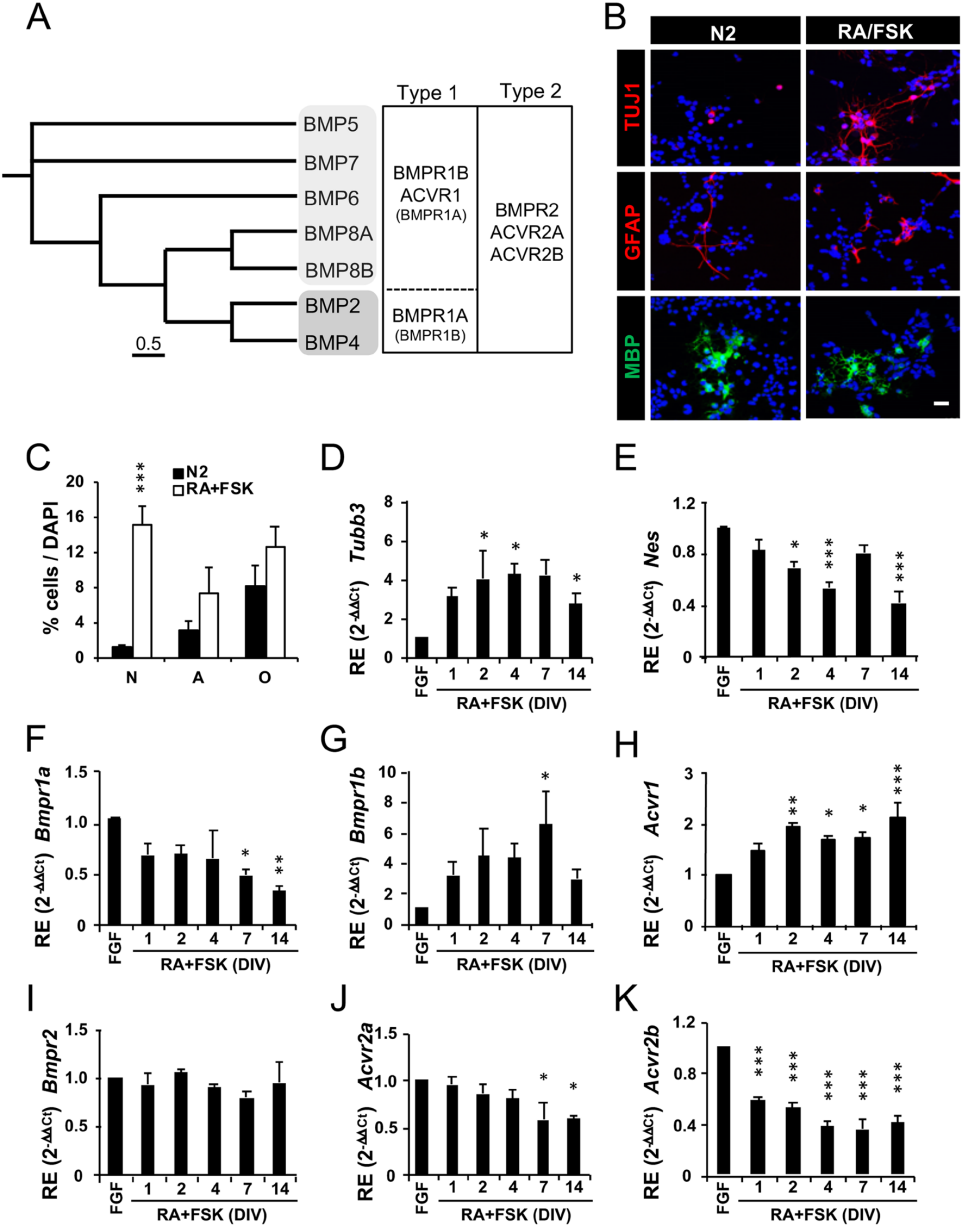
Expression of BMP ligands and BMP receptors in the adult hippocampal dentate gyrus and in AH-NSPCs undergoing differentiation. (**A**) Phylogenetic Tree showing distances between the mouse BMPs (left). The tree was generated using the alignment viewer ‘*AliView’*. The preferred type1/2 receptors bound by the different BMP ligands are also shown (right). (**B**–**C**) Differentiation of AH-NSPCs in control medium (N2) or N2 supplemented with Retinoic Acid (RA, 1 µM) and Forskolin (FSK, 5 µM) during 4 days *in vitro*. The percentage of neurons (N), astrocytes (A) and oligodendrocytes (O) was analysed by immunostaining against Tubulin βIII (Tuj1), Glial Fibrillary Acidic Protein (GFAP) and Myelin Basic Protein (MBP), respectively. The RA + FSK treatment favoured neuronal differentiation (average ± sem, n = 3, *two-tailed T-test:* ^***^P < *0*,*001*). (**D**–**K**) Relative gene expression (RE) patterns during the time course of AH-NSPC differentiation for *Tubb3* (**D**), *Nes* (**E**), *Bmpr1a* (**F**), *Bmpr1b* (**G**), *Acvr1* (**H**), *Bmpr2* (**I**), *Acvr2a* (**J**) and *Acvr2b* (**K**). *Sdha* was used as the housekeeping gene and expression levels were referred to those of proliferating AH-NSPCs grown in fibroblast growth factor 2 (FGF). Data correspond to average ± sem of n = 3 independent experiments analysed by the 2^−ΔΔCt^ method (*ANOVA:* ^*^P < *0*.*05;* ^**^P < *0*.*01;* ^***^P < *0*.*001)*. Scale bar in B, 25 µm.

Based on sequence similarity and function, BMP ligands can be subdivided into several subgroups that share a number of BMP type 1 and type 2 receptors (Fig. 1A). To gain a deeper understanding on their role in adult neurogenesis, we first examined their expression in the adult mouse hippocampal dentate gyrus (DG) and in cultured adult hippocampal neural stem and progenitor cells (AH-NSPCs) from rat by quantitative RT-PCR. On the basis of the C_t_ (cycle threshold) values, we found that most BMP ligands and receptors are expressed at the mRNA level in the adult DG (Supplementary Fig. S1).

We next evaluated the timing of BMP receptor expression in AH-NSPC cultures undergoing differentiation. AH-NSPCs were induced to differentiate for up to 14 days *in vitro* (DIV) using a combination of Retinoic Acid (RA) and Forskolin (FSK), a well-established treatment that facilitates the acquisition of the neuronal fate (Fig. 1B,C; Hsieh *et al*., 2004). After RNA extraction and cDNA synthesis, the gene expression pattern of the BMP type 1 receptors (*Bmpr1a*, *Bmpr1b* and *Acvr1*) and the BMP type 2 receptors (*Bmpr2*, *Acvr2a* and *Acvr2b*) was determined by quantitative RT-PCR and referred to the housekeeping genes *Sdha* (Fig. 1) and *18 S* (Supplementary Fig. S2). Increased expression of the neuronal cytoskeleton gene βIII-tubulin (*Tubb3*) and decreased expression of the immature intermediate filament gene Nestin (*Nes*) were used to confirm the differentiation of the cultures (Fig. 1D,E). Our results showed a decrease in the expression of *Bmpr1a* and an increase in the expression of *Bmpr1b* and *Acvr1* as the cells differentiate (Fig. 1F–H). Expression of *Bmpr2* at the mRNA level remained relatively constant during the process whereas *Acvr2a* and *Acvr2b* expression progressively decreased (Fig. 1I–K). This regulated gene expression profile of the type 1 and type 2 receptors suggests an early role for BMPR1A, BMPR2, ACVR2A, ACVR2B and a late role for BMPR1B, ACVR1, BMPR2 during adult hippocampal neurogenesis.

### BMP2 and BMP4 are pro-neurogenic in AH-NSPCs

**Figure 2:**
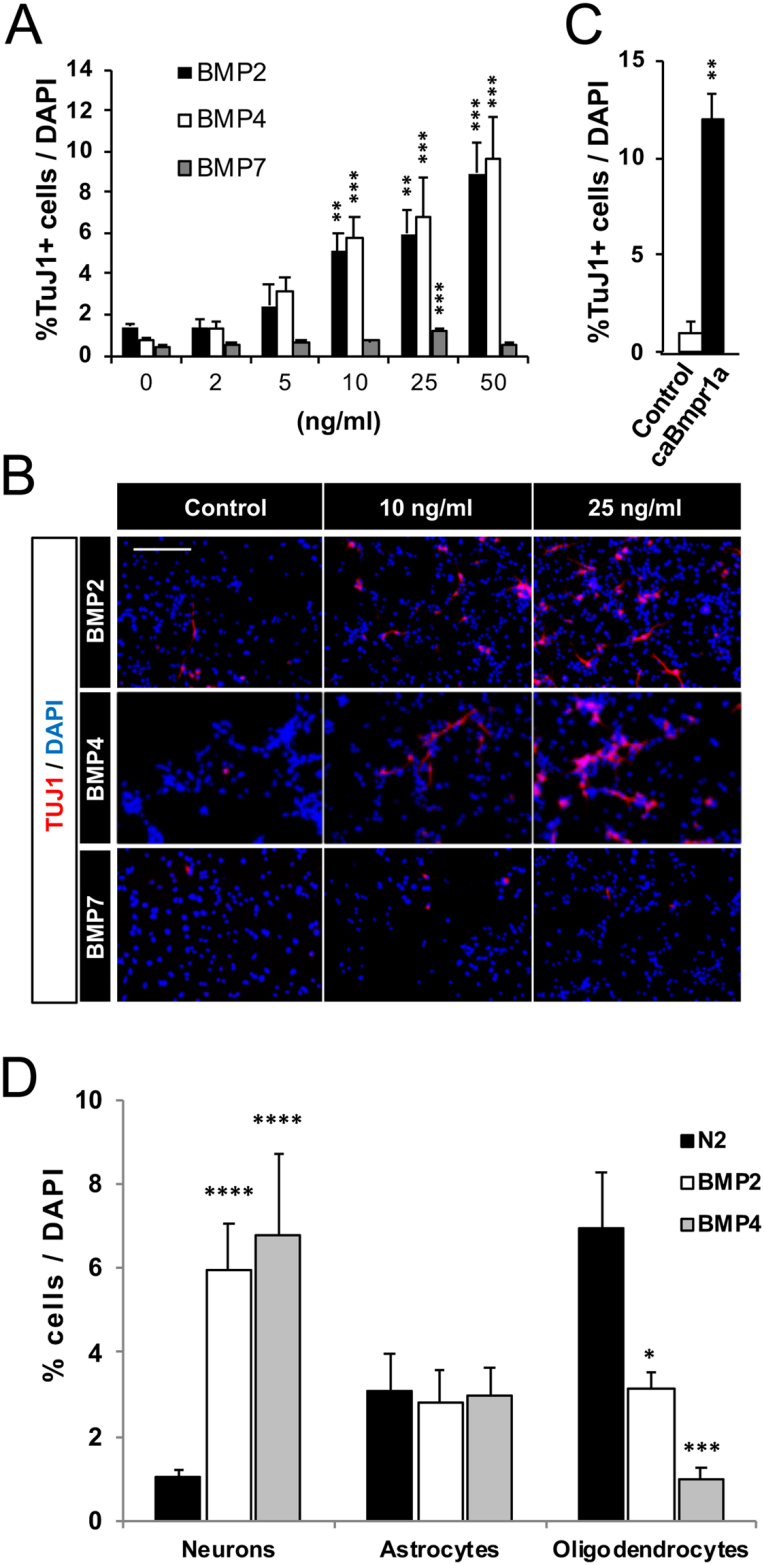
BMP2, BMP4 and caBMPR1A increase neurogenesis in AH-NSPC differentiation assays. (**A**) Percentage of Tubulin βIII (Tuj1) positive cells out of the total number of cells differentiated from AH-NSPCs in the presence of increasing concentrations of BMP2, BMP4 or BMP7. Data correspond to the average ± sem of n = 3. (**B**) Immunofluorescence images showing the levels of Tuj1 positive cells (red) and DAPI stain (blue) at 0, 10 and 25 ng/ml of BMP2, BMP4 and BMP7. As illustrated, BMP2 and BMP4 (but not BMP7) markedly induce neurogenesis *in vitro* in a dose-dependent manner. (**C**) Percentage of Tuj1 positive cells out of the total number of cells differentiated from AH-NSPCs transduced with a retroviral vector overexpressing a constitutive active form of BMPR1A (caBmpr1a) or with an empty vector as a control. (**D**) Percentage of neurons (N, Tubulin βIII positive cells), astrocytes (A, Glial Fibrillary Acidic Protein, GFAP positive cells) and oligodendrocytes (O, Myelin Basic Protein, MBP positive cells) in the absence (N2) or presence of 25 ng/ml of BMP2 or BMP4. The cell lineage analysis shows an increase in neurons and a decrease in oligodendrocytes in the presence of BMPs. Scale bar on B, 100 µm. ^*^P < *0*.*05;* ^**^P < *0*.*01;* ^***^P < *0*.*001*, ^****^P < *0*.*0001 by ANOVA*.

Since AH-NSPCs express different receptor combinations throughout differentiation, we next analysed whether BMP ligands with different affinities for those receptors influence neurogenesis. To this end, we cultured AH‐NSPCs in the absence of mitogenic stimulation but in the presence of an increasing concentration of several prototypic BMP ligands: BMP2 and BMP4 recombinant proteins, belonging to the Dpp subgroup that preferentially bind to BMPR1A; and BMP7 recombinant protein, belonging to the 60 A family that is best bound by BMPR1B and ACVR1^14,15^. After 4 DIV, neurogenesis was measured by immunostaining against the neuronal marker βIII‐tubulin (TuJ1). Our data show that BMP2 and BMP4 induce a marked dose-dependent increase in the percentage of TuJ1^+^ neurons, while BMP7 has a very mild effect (Fig. 2A,B). BMP2/4 also increased the number of neurons expressing the immature neuronal marker doublecortin (DCX) and the more mature neuronal marker MAP2 (25 ng/ml BMP4: 6.7 ± 1.1% MAP2^+^ cells, avergage ± sem, n = 3) compared to the control condition (N2 medium: 0.4 ± 0.2% MAP2^+^ cells, avergage ± sem, n = 3, *P* < 0.01, two-tailed T-test; Supplementary Fig. S3). Western blot analysis showed increased protein levels of βIII-tubulin and doublecortin (DCX) after BMP2/4 exposure (Supplementary Fig. S4), indicating altogether that ligands of the BMPR1A receptor such as BMP2/4 may be involved in instructing a neuronal cell fate *in vitro*. In support of this view, transduction of AH-NSPCs with a retroviral vector overexpressing a constitutive active form of BMPR1A mimicked the pro-neurogenic effect triggered by BMP2/4 (Fig. 2C). In addition, we found a reduction in oligodendrocytes in cultures treated with BMP2/4 but no change in the number of astrocytes (Fig. 2D and Supplementary Fig. S3), suggesting that BMP2/4 induce neurogenesis while decreasing oligodendrogenesis. Of note, a large fraction of the cells in the culture remained undifferentiated at 4 DIV (28.4 ± 2.4% of the cells were SOX2^+^ stem/progenitors in 25 ng/ml BMP4 *vs*. 62.6 ± 5.2% in control N2 medium, avergage ± sem, n = 3, *P* < 0.01, two-tailed T-test). Out of the SOX2^+^ cells, only 16.4 ± 3.7% were cycling (Ki67^+^) in the presence of BMP4, suggesting that most of the AH-NSPCs that did not engage in the differentiation programme stayed quiescent^19^.

**Figure 3:**
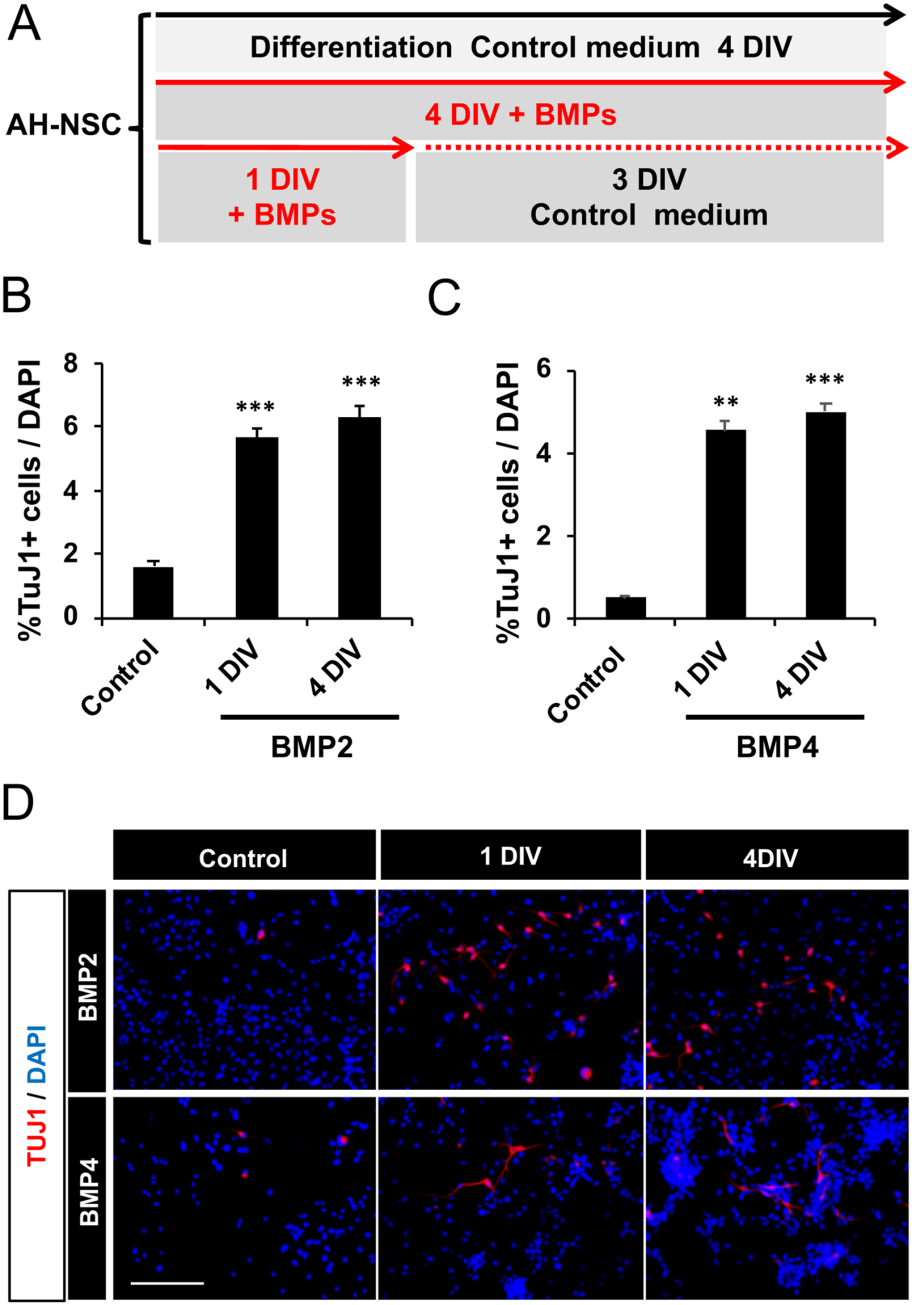
BMP2 and BMP4 influence the neuronal fate choice decision of the AH-NSPC progeny at an early timepoint. (**A**) Diagram describing the procedure. AH-NSPCs were stimulated transiently during 1 DIV or continuously during 4 DIV with 10 ng/ml of BMP2 or BMP4. Cells were fixed at 4 DIV and neurogenesis was measured by immunostaining against Tubulin βIII (Tuj1). (**B**,**C**) Percentage of Tuj1 positive cells after stimulating for 1 or 4 DIV with BMP2 (**B**) and BMP4 (**C**). Data correspond to the average ± sem of n = 3 independent experiments. (**D**) Immunofluorescence images showing the levels of Tuj1 positive cells (red) and DAPI stain (blue) at 4DIV in cultures stimulated transiently (1 DIV) or continuously (4 DIV) with BMP2 or BMP4. Scale bar in D, 100 µm. ^**^P < *0*.*01;* ^***^P < *0*.*001 by two-tailed T-test*.

Since *Bmpr1a* is markedly expressed in undifferentiated AH‐NSPCs and decreases throughout the neuronal differentiation process^19^ (Fig. 1F), we hypothesized that BMP2/4 would be acting very early in the neurogenic lineage, influencing the cell fate choice decision of the AH-NSPC progeny. To test this idea, we transiently exposed AH-NSPCs to BMP2 or BMP4 for 24 hours (1 DIV) and then cultured the cells for 3 additional days in the absence of BMP stimulation (Fig. 3A). We found that AH‐NSPCs exposed to BMP2 or BMP4 for 1 DIV reached similar neuronal differentiation levels than AH-NSPCs cultured for 4 DIV in the continuous presence of BMPs (Fig. 3B–D). These data show that a brief BMP2/4 treatment during the initial stages of differentiation is sufficient to promote neurogenesis and indicate that BMP2/4 can specify the neuronal fate of AH‐NSPCs within the first 24 hours of the differentiation time course.

### BMP2 and BMP4 induce neurogenesis through the activation of the BMP canonical pathway

**Figure 4:**
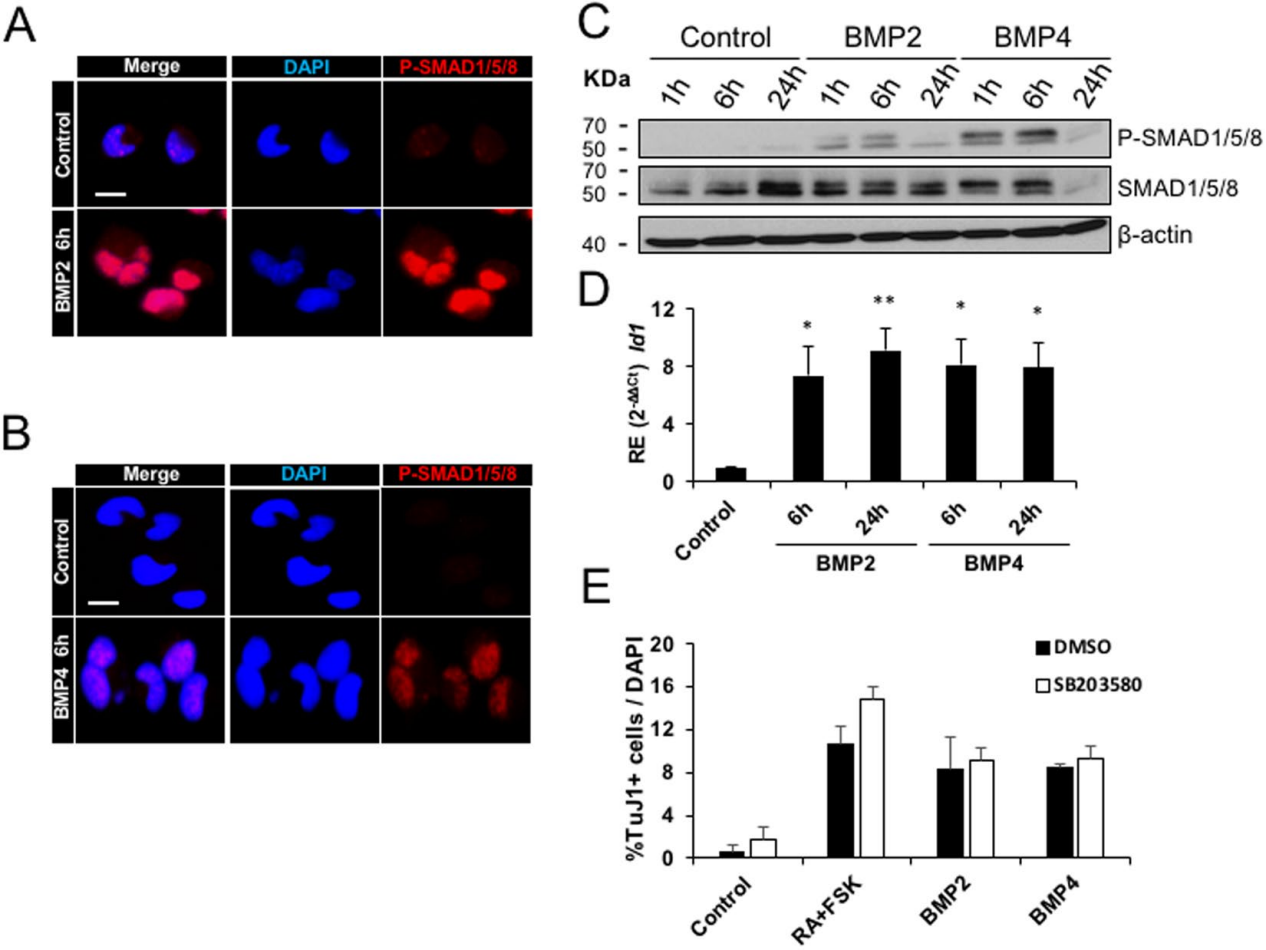
BMP2 and BMP4 induce neurogenesis through the activation of the P-SMAD canonical pathway in AH-NSPCs. (**A**,**B**) Immunofluorescence images of AH-NSPCs showing the increase in P-SMAD1/5/8 levels and the nuclear translocation of the phosphorylated proteins upon BMP2 stimulation (**A**) or BMP4 stimulation (**B**) for 6 hours. (**C**) Western immunoblot of whole cell lysates from AH-NSPCs treated with BMP2 or BMP4, separated by SDS-PAGE and blotted sequentially with antibodies against P-SMAD1/5/8, total SMAD1/5/8 and β-actin as loading control. Full-length blots are included in Supplementary Fig. S5. (**D**) Relative gene expression (RE) patterns for the canonical BMP pathway target gene *Id1* upon BMP2 or BMP4 stimulation at the indicated time points. *Sdha* was used as the housekeeping gene and expression levels were referred to untreated AH-NSPCs (Control). Data correspond to average ± sem of n = 3 independent experiments analysed by the 2^−ΔΔCt^ method (*two-tailed T-test:* ^*^P < *0*.*05;* ^**^P < 0.0*1)*. (**E**) Percentage of Tubulin βIII (Tuj1) positive cells out of the total number of cells differentiated from AH-NSPCs during 4 days *in vitro* in the presence of Retinoic Acid (RA, 1 µM) and Forskolin (FSK, 5 µM), BMP2 (25 ng/ml) or BMP4 (25 ng/ml) and P38MAPK inhibitor SB203580 or DMSO as a control. Data correspond to the average ± sem of n = 3. Inhibition of the non-canonical BMP signalling employing SB203580 had no effect on the increase in neurogenesis. Scale bar in A and B, 100 µm.

We next wanted to check whether BMP2/4 signal through the SMAD-dependent canonical pathway. AH-NSPCs were treated with BMP2 or BMP4 and were then analysed at different time points by immunofluorescence, Western blot and quantitative RT-PCR. As shown in Fig. 4, BMP2 and BMP4 rapidly triggered the phosphorylation and nuclear translocation of SMAD1/5/8 (Fig. 4A–C) and upregulated the expression of the SMAD target gene *Id1* (Fig. 4D and Supplementary Fig. S3) confirming the activation of the canonical pathway. In agreement with this finding, inhibition of non-canonical BMP signalling employing the P38MAPK inhibitor SB203580 had no effect on the increase in neurogenesis induced by BMP2/4 exposure (Fig. 4E). Phosphorylation of SMAD1/5/8 was transient and decreased after the initial 24 hours of stimulation with the BMP ligands, indicating that AH-NSPCs did not maintain sustained activation of the BMP canonical pathway during the 4 DIV differentiation period (Fig. 4C and Supplementary Fig. S6). This further reinforces the view that the signalling inducing the neuronal fate occurs in the first 24 hours (1 DIV) of the differentiation process (Fig. 3). Together, the data indicate that BMP2 and BMP4 enhance neuronal production through the transient activation of the P-SMAD1/5/8-dependent canonical signalling pathway.

### BMP2/4 canonical signalling synergizes with WNT canonical signalling to increase neurogenesis

It has been previously reported that adult hippocampal neurogenesis is tightly regulated by the canonical WNT/β-catenin signalling pathway, both *in vitro* and *in vivo* [reviewed in^25^], and that WNT3/WNT3A ligands markedly enhance the neuronal differentiation of AH-NSPCs^10,26^. Given the phenotypic similarity resulting from the activation of the BMP and WNT pathways, we searched for a possible crosstalk between the two.

**Figure 5:**
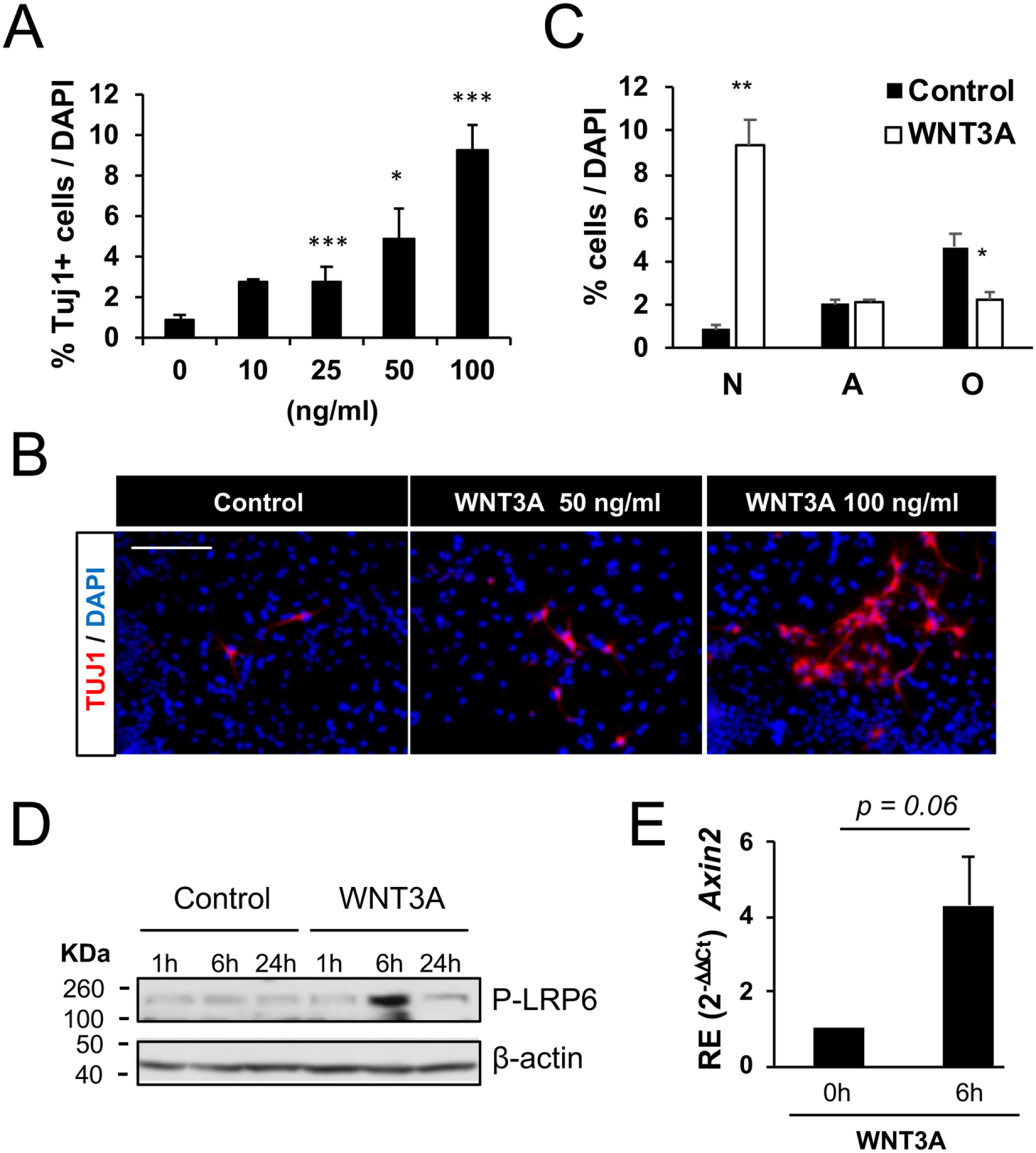
WNT3A increases neurogenesis in AH-NSPC differentiation assays. (**A**) Percentage of Tubulin βIII (Tuj1) positive cells out of the total number of cells differentiated from AH-NSPCs in the presence of increasing concentrations of WNT3A. Data correspond to the average ± sem of n = 3 (^*^P < 0.*05;* ^***^P < *0*.*001 by ANOVA)*. (**B**) Immunofluorescence images showing the levels of Tuj1 positive cells (red) and DAPI stain (blue) at 0, 50 and 100 ng/ml of WNT3A. As illustrated, WNT3A induces neurogenesis *in vitro* in a dose-dependent manner. (**C**) Percentage of neurons (N, Tubulin βIII positive cells), astrocytes (A, Glial Fibrillary Acidic Protein, GFAP positive cells) and oligodendrocytes (O, Myelin Basic Protein, MBP positive cells) in the absence (N2) or presence of 100 ng/ml of WNT3A. The cell lineage analysis shows an increase in neurons and a decrease in oligodendrocytes in the presence of WNT3A (^*^P < *0*.*05;* ^**^P < *0*.*01 by ANOVA)*. (**D**) Western immunoblot of whole cell lysates from AH-NSPCs treated with WNT3A (100 ng/ml), separated by SDS-PAGE and blotted sequentially with antibodies against P-LRP6 and β-actin as loading control. Full-length blots are included in Supplementary Fig. S7. (**E**) Relative gene expression (RE) pattern for the WNT pathway target gene *Axin2* upon WNT3A (100 ng/ml) stimulation at 6 h. *Sdha* was used as the housekeeping gene and expression levels were referred to untreated AH-NSPCs (0 h). Data correspond to average ± sem of n = 3 independent experiments analysed by the 2^−ΔΔCt^ method (*two-tailed T-test: P* = *0*.*06)*. Scale bar in B, 100 µm.

To this end, we first exposed AH-NSPCs to increasing amounts of the WNT3A ligand and confirmed that recombinant WNT3A increased the number of neurons at 4DIV in a dose-dependent manner (Fig. 5A,B). Interestingly, as with BMP2/4, the acquisition of the neuronal fate in the presence of WNT3A was also accompanied by a reduction in the oligodendroglial fate (Fig. 5C). In addition, we confirmed that WNT3A triggered the canonical pathway in AH-NSPCs, since it increased the phosphorylation of LRP6 (Fig. 5D) and the expression of the *Axin2* gene (Fig. 5E).

**Figure 6:**
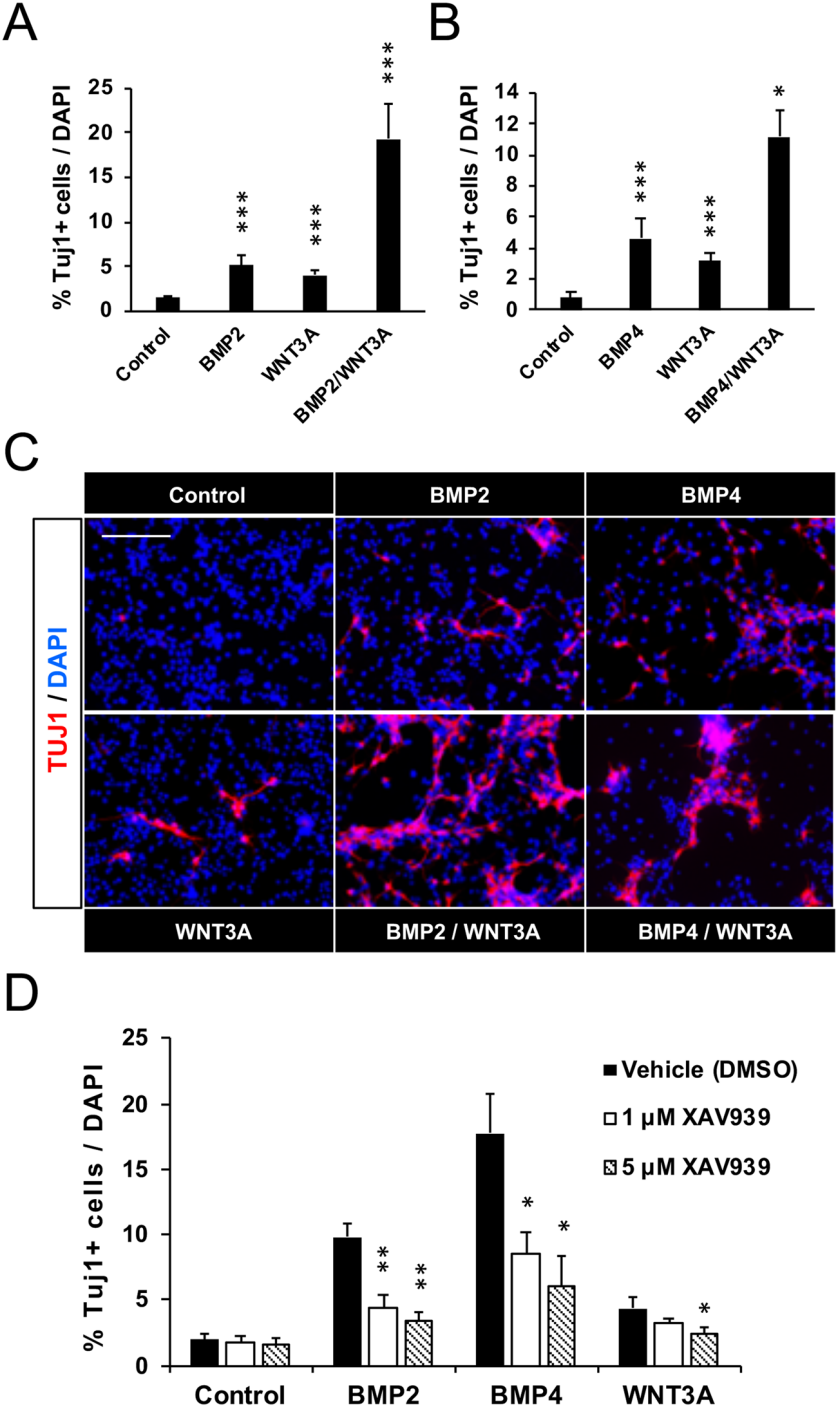
BMP2/4 require endogenous WNT signalling and synergize with exogenous WNT3A to increase neurogenesis in AH-NSPC differentiation assays. (**A**) Percentage of Tubulin βIII (Tuj1) positive cells out of the total number of cells differentiated from AH-NSPCs during 4 days *in vitro* in the presence of BMP2 (25 ng/ml), WNT3A (25 ng/ml) or a combination of both BMP2 and WNT3A. Data correspond to the average ± sem of n ≥ 5 (2-way *ANOVA:* ^***^P < *0*.*001)*. (**B**) Percentage of Tubulin βIII (Tuj1) positive cells out of the total number of cells differentiated from AH-NSPCs during 4 days *in vitro* in the presence of BMP4 (25 ng/ml), WNT3A (25 ng/ml) or a combination of both BMP4 and WNT3A. Data correspond to the average ± sem of n ≥ 6. (2-way *ANOVA:* ^*^P < *0*.*05;* ^***^P < *0*.*001)*. (**C**) Immunofluorescence images showing the levels of Tuj1 positive cells (red) and DAPI stain (blue) at the indicated conditions. As illustrated, the number of neurons in the combined BMP2/4 + WNT3A treatment is higher than in the BMP2/4 or WNT3A independent treatments, evidencing a synergistic effect in neurogenesis of the BMP and WNT pathways. (**D**) Percentage of Tubulin βIII (Tuj1) positive cells out of the total number of cells differentiated from AH-NSPCs during 4 days *in vitro* in the presence of BMP2 (50 ng/ml), BMP4 (50 ng/ml) or WNT3A (50 ng/ml) and XAV939 (a compound that blocks Wnt/b-catenin activity through the stabilization of Axin via tankyrase inhibition) or DMSO as a control. Data correspond to the average ± sem of n = 3 (*two-tailed T-test:* ^*^P < *0*.*05;* ^**^P < *0*.*01)*. Scale bar in B, 100 µm.

Having verified the pro-neurogenic effect of WNT3A, we next exposed AH-NSPCs to low doses of the BMP2/4 and WNT3A ligands separately or in combination to test for a possible interaction between the two pathways (Fig. 6A–C). Statistical analysis by 2-way ANOVA revealed a significant synergistic effect in neurogenesis of the BMP and WNT factors (F_1,28_ = 14.642, *P* < 0.001). At BMP2 and WNT3A concentrations of 25 ng/ml, a marked raise in TuJ1^+^ cells was elicited with a 19-fold increase in the number of neurons when both ligands were present, relative to the 5- and 3.5-fold increases elicited by BMP2 or WNT3A treatments alone, respectively (Fig. 6A). A similar profile was obtained for BMP4 and WNT3A (F_1,30_ = 5.851, *P* < 0.05, Fig. 6B).

### The pro-neurogenic BMP2/4 activity requires endogenous canonical WNT signalling and is linked to LEF1 expression

Given that WNT signalling is absolutely required for neurogenesis to proceed in the adult hippocampus^25^, we speculated that, not only the BMP and WNT pathways synergize when both ligands are added exogenously to the AH-NSPC culture, but that BMP signalling requires a basal level of WNT signalling to exert its pro-neurogenic effect. In other words, we hypothesized that the BMP treatment alone could be potentiating endogenous WNT signalling. To test for this hypothesis, we differentiated AH-NSPCs in the presence of BMP2/4 (50 ng/ml) concomitant with increasing concentrations of the canonical WNT signalling inhibitor XAV939^27^ to prevent endogenous canonical WNT signalling (Fig. 6D). Blockade of the signalling elicited by exogenously added WNT3A (50 ng/ml) was used as a control for the XAV939 treatment. Interestingly, we found that endogenous WNT signalling is required at least in part for the neurogenic action of the BMP2/4 ligands.

**Figure 7:**
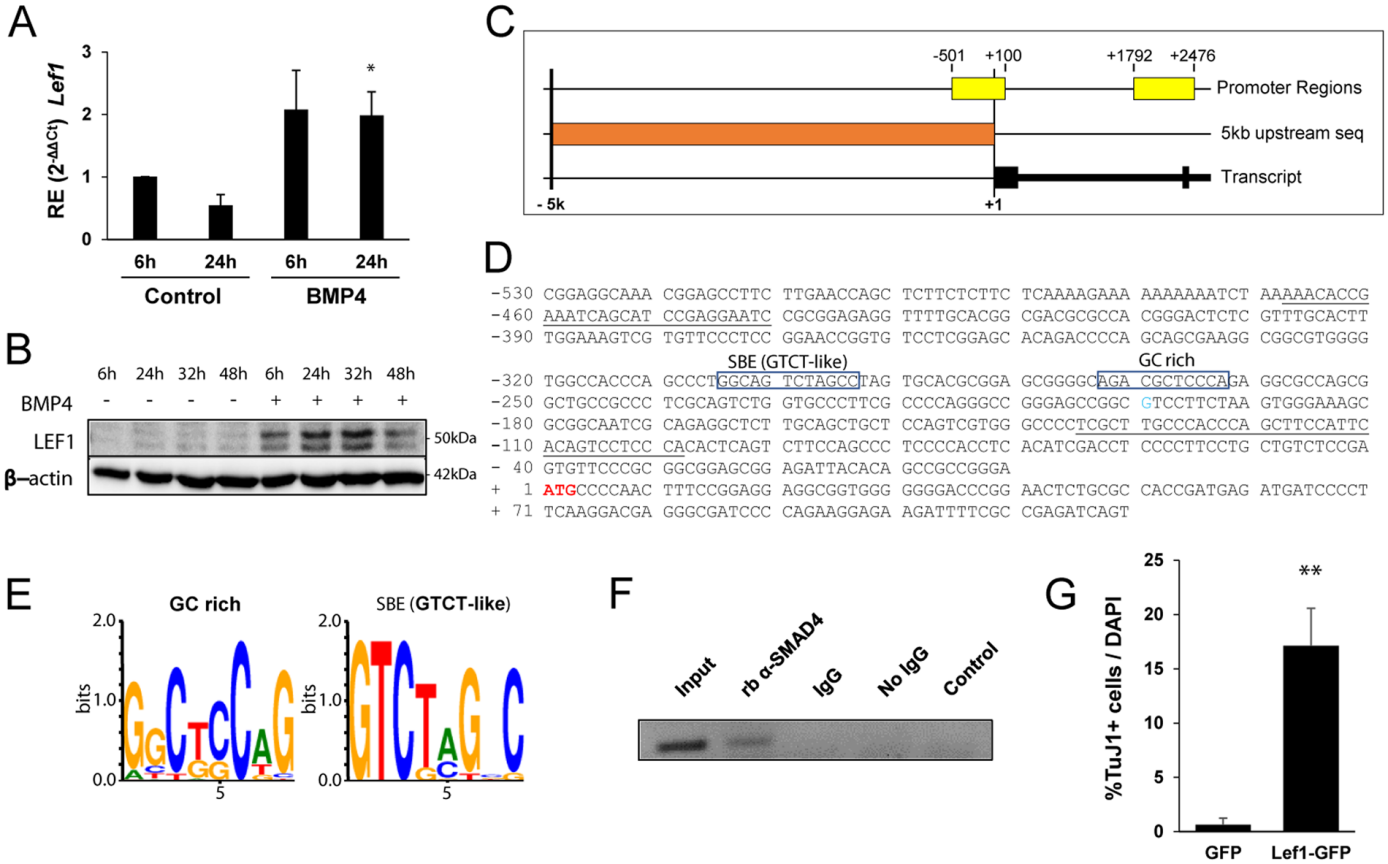
Lef1 is a direct target of BMP4 signalling in AH-NSPCs. (**A**) Relative gene expression (RE) pattern for the *Lef1* gene upon BMP4 (50 ng/ml) stimulation at the indicated time points. *Sdha* was used as the housekeeping gene and expression levels were referred to untreated AH-NSPCs (Control). (**B**) Western immunoblot of whole cell lysates from AH-NSPCs treated with BMP4 at the indicated time points, separated by SDS-PAGE and blotted sequentially with antibodies against LEF1 and β-actin as loading control. Full-length blots are included in Supplementary Fig. S8. (**C**) *Lef1* promoter region analysis using *Genomatix Promoter Inspector* software. Image shows the retrieved 5 kb sequence (orange) upstream of the *Lef1* coding sequence (black) and two validated promoter regions that result from the promoter analysis (yellow). (**D**,**E**) Nucleotide sequence upstream of the *Lef1* translational start site (ATG, red). Two SMAD transcription factor binding sites were predicted: the first one, positioned at −274/−264, corresponds to a SMAD Binding Element (SBE) (GTCT-like), and the second one, positioned at −233/−223, corresponds to a GC rich element. (**F**) ChIP assay of BMP4-treated AH-NSPCs using a SMAD4 rabbit antibody. For the PCR amplification of the precipitated material, the underlined primers in (**D**) were employed. Rabbit IgG (IgG) was used as a control. Full-length gel is included in Supplementary Fig. S9. (**G**) Percentage of Tubulin βIII (Tuj1) positive cells out of the total number of cells differentiated from AH-NSPCs transduced with a lentiviral vector overexpressing Lef1-GFP or GFP as a control. Cells were cultured during 4 days *in vitro*. ^*^P < *0*.*05;* ^**^P < *0*.*01 by two-tailed T-test*.

Canonical WNT signalling triggers the expression of pro-neurogenic genes through T-cell factor/lymphoid enhancer factor (TCF/LEF)-binding sites in their promoters^26^. It has been reported that the expression of *Lef1* can be activated by BMP4 in BMP-mediated inductive tissue interactions^28^, raising the possibility that the convergence between BMP2/4 and WNT3A signalling is located nearby LEF1. Thus, we next explored whether BMPs could increase *Lef1* expression in AH-NSPCs. As shown in Fig. 7A, following a 6-hour or 24-hour treatment with BMP4, *Lef1* mRNA levels were increased. This resulted in the accumulation of LEF1 protein (Fig. 7B).

We next analysed the *Lef1* gene promoter region to check for the presence of conserved BMP regulatory elements. We searched the rat genome databases, retrieved the 5 kb sequence upstream of the initial *Lef1* ATG start codon located in Chr2 and performed a theoretical analysis of the sequence using promoter inspector tools. We found a validated TATA-less promoter sequence spanning from −501 to +100 relative to the translational start site ATG codon at +1 (601 bases in length, P1) and a second theoretical promoter region (684 bases in length, P2) within the first intron of the *Lef1* gene (Fig. 7C). The structure of the rat *Lef1* gene resembles that of the human *LEF1* gene, a multipromoter gene characterized by a first 5′ promoter (P1) lacking a consensus TATA box and a second intronic promoter (P2) located downstream of exon 1^29,30^. In human, the P1 promoter drives the expression of a full-length LEF1 polypeptide; the P2 promoter instead produces a truncated protein that lacks the beta-catenin binding domain and supresses WNT signalling^30^. From these two promoter sequences, we selected the upstream −501/+100 P1 promoter for further analysis and scanned the sequence for transcription factor binding sites (TFBS). Out of the 154 total TFBS sites mapped to the proximal upstream promoter region, two corresponded to canonical SMAD protein binding sites. The SMAD binding sites were located at position −302 (“5′-GTCT-3′ SBE” or SMAD4 binding element) and −261 (“GCCG-like motif” or GC-rich SMAD1/5 binding element) from the start codon, and were recognized as putative binding sites for P-SMAD/SMAD4 dimers (Fig. 7D,E). To investigate the direct interaction between SMAD proteins and the *Lef1* promoter, we performed a Chromatin Immunoprecipitation (ChIP) assay with anti-SMAD4 antibodies, followed by PCR analysis with primer pairs amplifying the region containing the putative SMAD binding sites (Fig. 7D). As shown in Fig. 7F, the ChIP-PCR results revealed the presence of SMAD4 on the *Lef1* gene promoter region comprised between −361 and −625 nucleotides in AH-NSPCs exposed to BMP4. Thus, our results show that in response to BMP stimulation SMAD4 associates with the DNA at the *Lef1* proximal promoter region, which includes the predicted SMAD binding elements. The binding correlates with an increase in *Lef1* mRNA levels (Fig. 7A). Finally, we found that transduction of AH-NSPCs with a retroviral vector overexpressing *Lef1* is sufficient to enhance neuronal production even in the absence of exogenous WNT stimulation (Fig. 7G), thereby mimicking the BMP2/4 effect.

Altogether, the data indicate that *Lef1* is a direct target gene of SMAD4 and suggest that the increase in the expression of the transcription factor LEF1 downstream of BMP signalling may be potentiating adult hippocampal neurogenesis.

## Discussion

**Figure 8:**
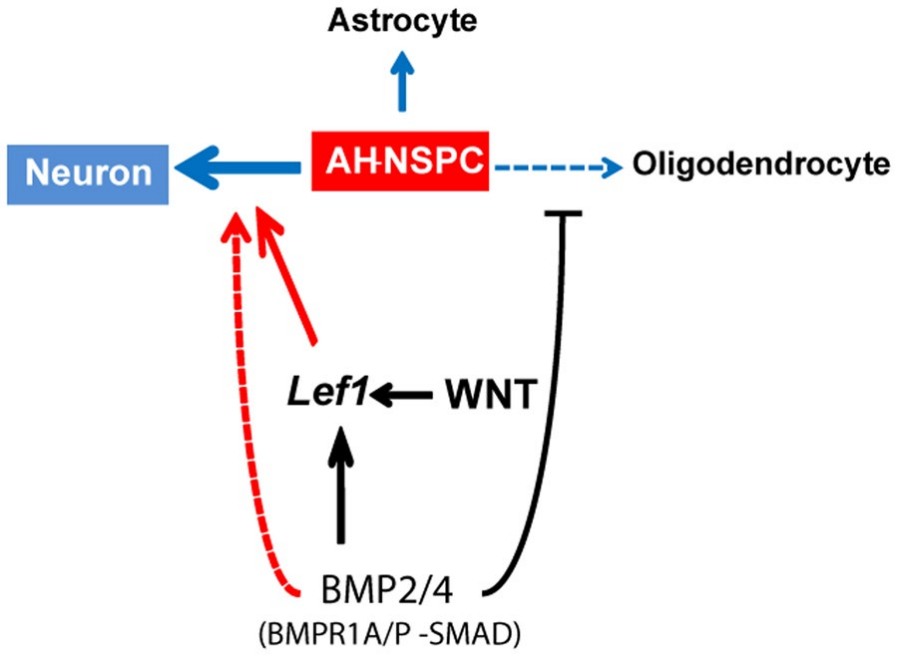
BMP2/4 increase neurogenesis from adult hippocampal NSPCs and synergize with WNT3A. Schematic of our proposed model summarizing the main results. BMP2/4 induce neurogenesis through the activation of the P-SMAD canonical pathway downstream of the BMPR1A type 1 receptor. The pro-neurogenic effect of BMP signalling is partly dependent on endogenous WNT signalling, and the mechanism relies on the up-regulation of the *Lef1* gene, a direct SMAD target. WNT-independent effects of the BMPs are not excluded (dashed line, red). BMP2/4 also decrease the number of oligodendrocytes.

It has been previously reported that neurogenesis in the adult mammalian brain is dynamically regulated by a number of local signals from the neural stem cell microenvironment, including BMPs and WNTs. High levels of canonical BMP signalling have been detected in the hippocampal niche, the majority of the stem and progenitor cells showing phosphorylated SMAD1/5/8 proteins in the nucleus^19,20^. Moreover, ligands from the BMP family have been involved in the regulation of adult hippocampal stem cell quiescence^19,20^ and in the control of progenitor maturation at multiple stages along the neurogenic lineage^18^. However, the contribution of the BMP pathway to the fate specification of the hippocampal neural stem cell progeny during adulthood, in concert with other key niche signals, has remained largely unexplored. The *in vitro* data included herein give further insight into the extensive role of BMP molecules as regulators of stem cell differentiation in the adult hippocampus, and support a model whereby BMPs would have WNT-dependent instructive effects, favouring the acquisition of the neuronal fate possibly at the expense of the oligodendroglial fate (Fig. 8). This lines up with the early pro-neurogenic role of BMP signalling during the neurogenic phases of forebrain development^31,32^.

BMP signalling has been previously shown to promote an astrocytic fate in embryonic subventricular zone multipotent NSPC mouse cultures grown in EGF^33,34^. In our *in vitro* setup, however, BMP2/4 did not change the astroglial fate of adult hippocampal NSPCs isolated from rat and expanded in FGF2. This inconsistency may be due to a difference in the origin of the cells, in the cell culture conditions or perhaps in their basal level of WNT signalling. Interestingly, *in vivo* it has been shown that increasing BMP4 levels in the adult mouse hippocampus, by means of injecting a lentivirus overexpressing BMP4, decreases neural stem/progenitor cell proliferation, delays maturation, but it does not increase the proportion of cells that differentiate into astroglia^18^. This suggests that under physiological conditions BMP may not be pro-gliogenic in the adult hippocampal niche. However, we cannot exclude pro-gliogenic effects of the BMP pathway upon sustained up-regulation of BMP signalling or under pathological conditions that lead to an increase in gliosis^33,35^.

As for the contribution of the specific subtypes of BMP receptors, the gene expression patterns obtained suggest that BMPR1A receptor has an early role in the differentiation process, while BMPR1B and ACVR1 may possibly have a late role. The early role of BMPR1A is consistent with the results obtained in the differentiation assays employing the preferred BMPR1A ligands (BMP2/4) and with the retroviral experiments in which we overexpressed a constitutive active form of BMPR1A. Moreover, we found that the differentiation levels attained under an initial and transient exposure to BMP2/4 were similar to those obtained with a continuous exposure to the ligands, further indicating that the signalling inducing the neuronal fate occurs in the first stages of the differentiation process and thus is likely transduced by the most highly expressed type 1 receptor in those initial stages, BMPR1A. Our results partly complement a previous study^36^, in which a dominant negative form of BMPR1A (dnBMPR1A) was overexpressed in AH-NSPCs. The dnBMPR1A, with a non-functional intracellular domain but a functional extracellular domain that traps the endogenously produced BMP ligands, increased astroglial differentiation of the cultures and this was likely mediated by the concomitant increase in the expression of BMPR1B in the cultures^36^.

To gain insight into potential mechanisms underlying the BMP pro-neurogenic action uncovered in our assay, we examined its dependency on WNT signalling, a master regulator of adult hippocampal neurogenesis^10^. We found that the BMP pro-neurogenic effect requires basal levels of endogenous WNT signalling and that the BMP and WNT pathway show a synergistic interaction. When combining the BMP and WNT ligands at non-saturating concentrations, the effect of the two taken together was greater than the sum of their separate effects at the same doses. In the light of these data, it seemed likely that the two signalling pathways would share components of the same mechanistic route.

The BMP and WNT pathways have been shown to interact with each other at multiple levels, for instance, via the binding of both SMAD4 and TCF/LEF transcription factors to the promoter and enhancer regions of certain target genes^37,38^. The TCF/LEF family of transcription factors mediate the downstream effects of canonical Wnt/β-catenin cascade and has four members in mammals, LEF1 being the most well characterized^39^. Given LEF1 is expressed in adult hippocampal neural stem cells and is required for neurogenesis^3,26,40^, and since the expression of *Lef1* can be activated by BMP4 in BMP-mediated inductive tissue interactions^28^, we focused on the *Lef1* gene as a possible convergence point between the BMP and the WNT routes. We identified functional BMP regulatory elements on the *Lef1* promoter, we found that *Lef1* expression was increased shortly after BMP exposure and we demonstrated that overexpressing *Lef1* is sufficient to enhance neurogenesis, uncovering a novel mechanistic link between the BMP/WNT signalling pathways operating in adult hippocampal neural stem and progenitor cells. A similar mechanism could be active as well during the development of the hippocampal dentate gyrus, since it has been recently reported that *Lef1* expression at embryonic stages is regulated by the BMP type 1 receptor ACVR1^41^.

In summary, signalling through the BMP type 1 receptor BMPR1A plays a relevant role in the modulation of adult hippocampal neurogenesis, regulating not only NSC quiescence^18–20^ but possibly also the early stages of the differentiation cascade. At least *in vitro*, the pro-neurogenic canonical WNT signalling interplays with, and is subjected to regulation by, BMP signalling. This may be achieved by increasing the expression of the transcription factor LEF1. Although future *in vivo* studies are needed to further evaluate the effect of BMP signalling in fate specification, our *in vitro* model already shows that the role of BMPs/BMPRs during adult neurogenesis is much more complex than was previously thought.

## Electronic supplementary material

Supplementary information
