# Supplementary material for: BMP and WNT signalling cooperate through LEF1 in the neuronal specification of adult hippocampal neural stem and progenitor cells
Source: Sci Rep. 2018 Jun 18;8:9241. doi: 10.1038/s41598-018-27581-0 (PMC6006330; doi:10.1038/s41598-018-27581-0)
Supplement: Supplementary file 1 — Supplementary information [file 41598_2018_27581_MOESM1_ESM.pdf]

SUPPLEMENTARY INFORMATION

BMP and WNT signalling cooperate through LEF1 in the neuronal specification of adult hippocampal neural stem and progenitor cells

Tomás Armenteros, Zoraida Andreu, Rafael Hortigüela, D. Chichung Lie & Helena Mira.

Gene	Ct DG	Ct AH-NSPCs
Bmp2	31.4±0.8	28.7±0.1
Bmp4	31.1±0.6	27.9±0.1
Bmp5	30.1±0.5	34.3±0.9
Bmp6	29.7±0.6	32.9±0.4
Bmp7	31.4±0.4	27.9±0.0
Bmp8a	n.d.	n.d.
Bmp8b	33.1±0.2	38.7±0.5
Bmpr1a	27.5±0.5	26.1±0.2
Bmpr1b	32.2±0.2	34.5±0.5
Bmpr2	23.5±0.3	23.1±0.0
Acvr1	26.0±0.2	29.4±0.1
Acvr2a	25.1±0.2	25.5±0.0
Acvr2b	26.9±0.3	24.8±0.0

Supplementary Fig. S1. BMP ligands and receptors are expressed in the Dentate Gyrus of adult mice (DG) and in rat AH-NSPCs. Expression at the mRNA level was confirmed by quantitative RT-PCR using the corresponding primers for each gene (Ct, threshold cycle; average±sem, n=3). Transcripts for *Bmp8a* were not detected (n.d.).

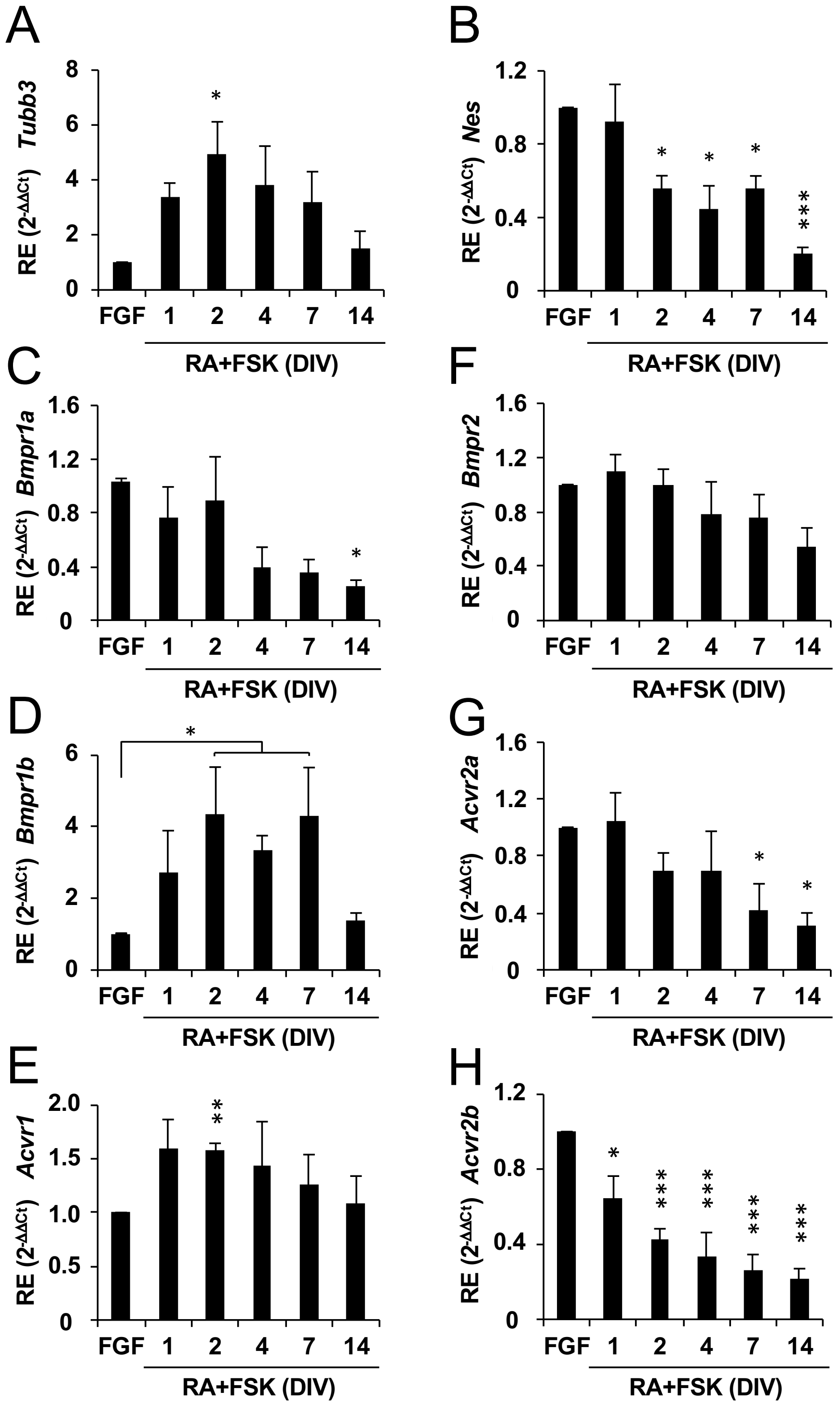

Supplementary Fig. S2. Expression of the BMP receptors during AH-NSPC differentiation. Relative gene expression (RE) patterns during the time course of AH-NSPC differentiation in N2 medium supplemented with Retinoic Acid (RA, 1µM) and Forskolin (FSK, 5µM) for *Tubb3* (**A**), *Nes* (**B**), *Bmpr1a* (**C**), *Bmpr1b* (**D**), *Acvr1* (**E**), *Bmpr2* (**F**), *Acvr2a* (**G**) and *Acvr2b* (**H**). The *18S* rRNA was used as the housekeeping gene and expression levels were referred to those of proliferating AH-NSPCs grown in fibroblast growth factor 2 (FGF). Data correspond to average±sem of n=3 independent experiments analysed by the $2^{-\Delta\Delta Ct}$ method (*ANOVA: * P < 0.05; ** P < 0.01; *** P < 0.001 for A-D and F-H; T-test: ** P < 0.01 for E).*

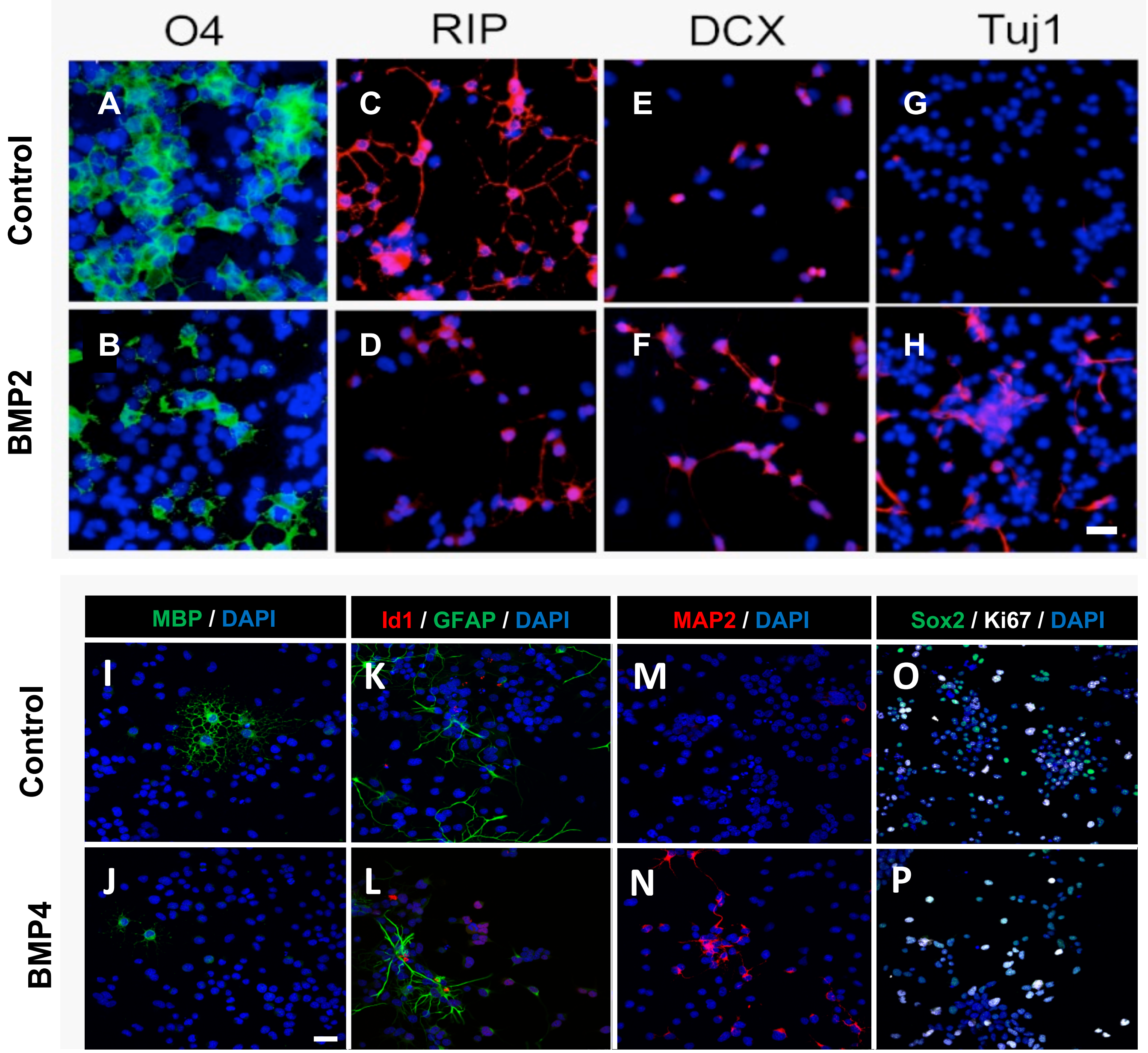

Supplementary Fig. S3. BMP2/4 increase neurogenesis and decrease oligodendrogenesis in AH-NSPC differentiation assays. *Upper pannel,* immunofluorescence images showing a reduction in the cells expressing the immature oligodendrocyte lineage markers O4 (**A** and **B**) and RIP (**C** and **D**), and an increase in the cells expressing the immature neuronal marker DCX (**E** and **F**) and the pan-neuronal marker βIII-tubulin (Tuj1, **G** and **H**). Scale bar, 25 μm. *Lower pannel, ,* immunofluorescence images showing a reduction in the cells expressing the mature oligodendrocyte lineage marker MBP (**I** and **J**) and an increase in the cells expressing Id1 (**K** and **L**) and the mature neuronal marker MAP2 (**M** and **N**). A reduction in the number of cells expressing the stem/progenitor marker Sox2 and the cell cycle marker Ki67 was also observed (**O** and **P**). Scale bar, 25 μm.

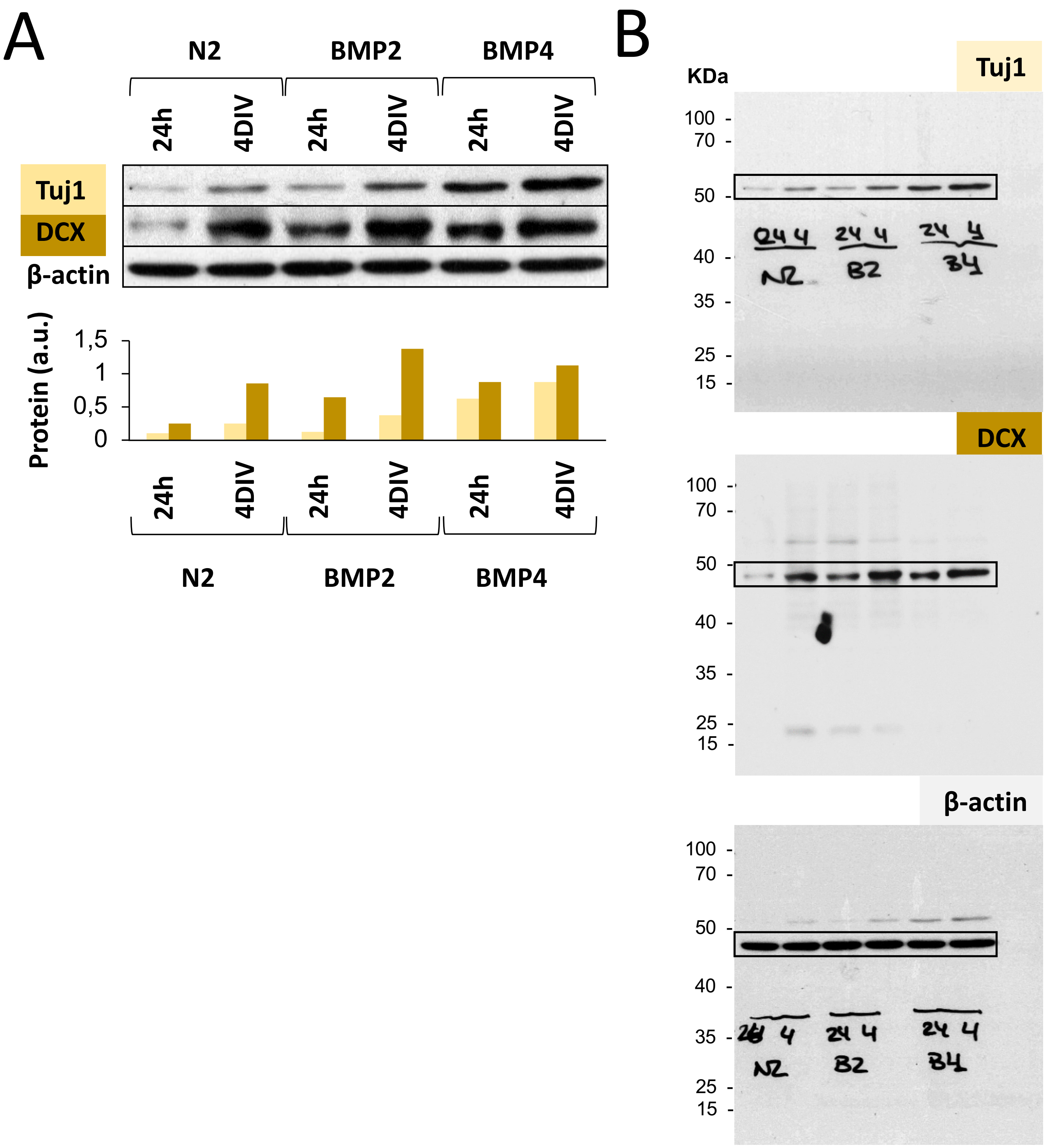

Supplementary Fig. S4. BMP2 and BMP4 increase the expression of neuronal markers in AH-NSPCs. (A) *Upper panel,* representative Western immunoblot showing the levels of βIII-tubulin (Tuj1) and the immature neuronal protein Doublecortin (DCX) in AH-NSPCs undergoing differentiation in N2 or N2 supplemented with 50 ng/ml of BMP2 or BMP4. Cells were lysed at 24 hours and 4 DIV of BMP2/4 exposure. β-actin was employed as the loading control. *Lower panel,* quantification for the Western blot shown above. Relative βIII-tubulin and DCX protein levels were normalized to β-actin (a.u., arbitrary units). **(B)** Original full-length Western immunoblots showing the cropped region used to compose the final image displayed in (A).

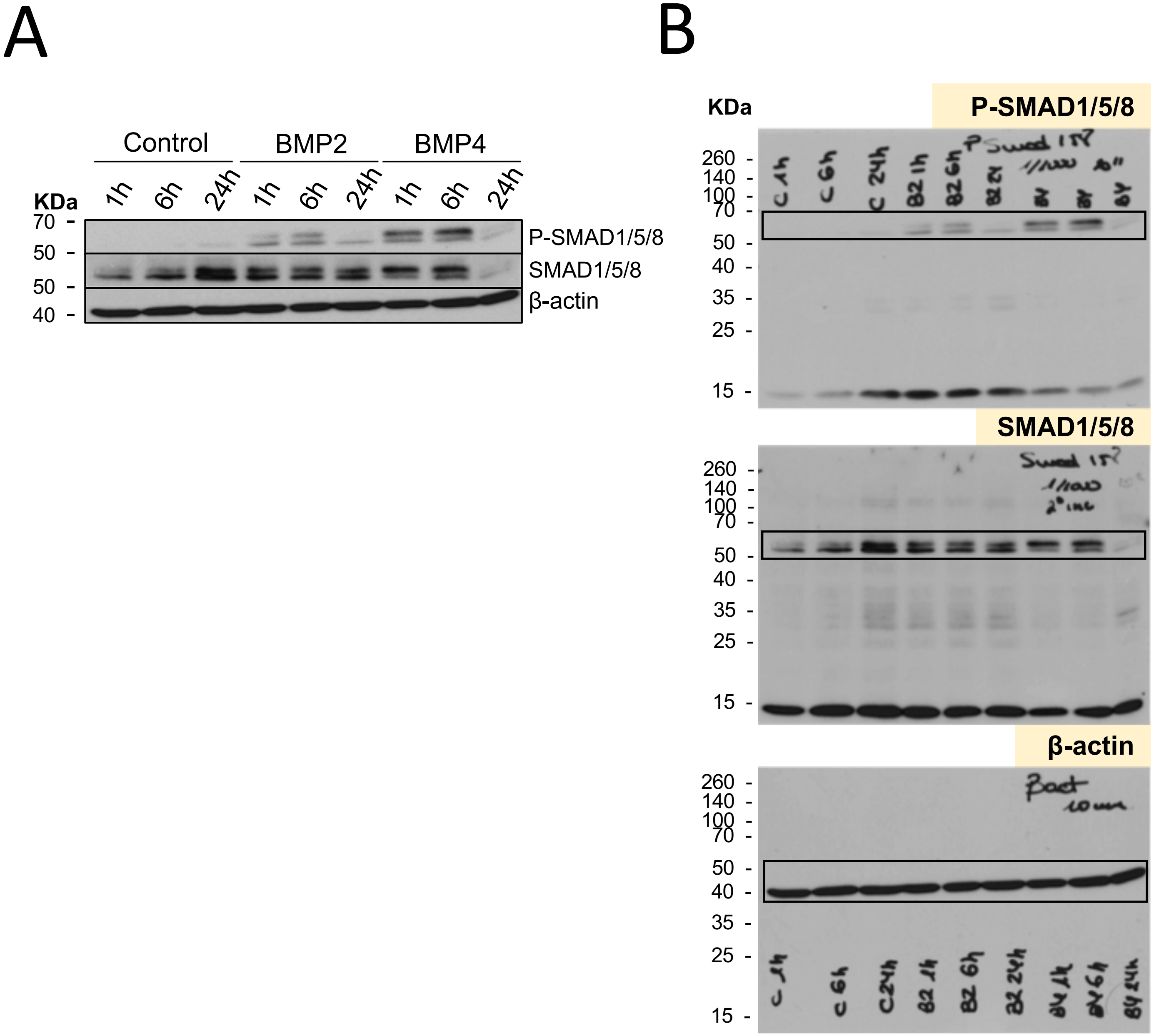

Supplementary Fig. S5. BMP2 and BMP4 activate the P-SMAD canonical pathway in AH-NSPCs. (A) Western immunoblot shown in Figure 4C. Whole cell lysates from AH-NSPCs treated with BMP2 or BMP4 were separated by SDS-PAGE and blotted sequentially with antibodies against P-SMAD1/5/8, total SMAD1/5/8 and β-actin as loading control. Data are representative for n=3 independent experiments. **(B)** Original full-length Western immunoblots showing the cropped region used to compose the final image displayed in (A).

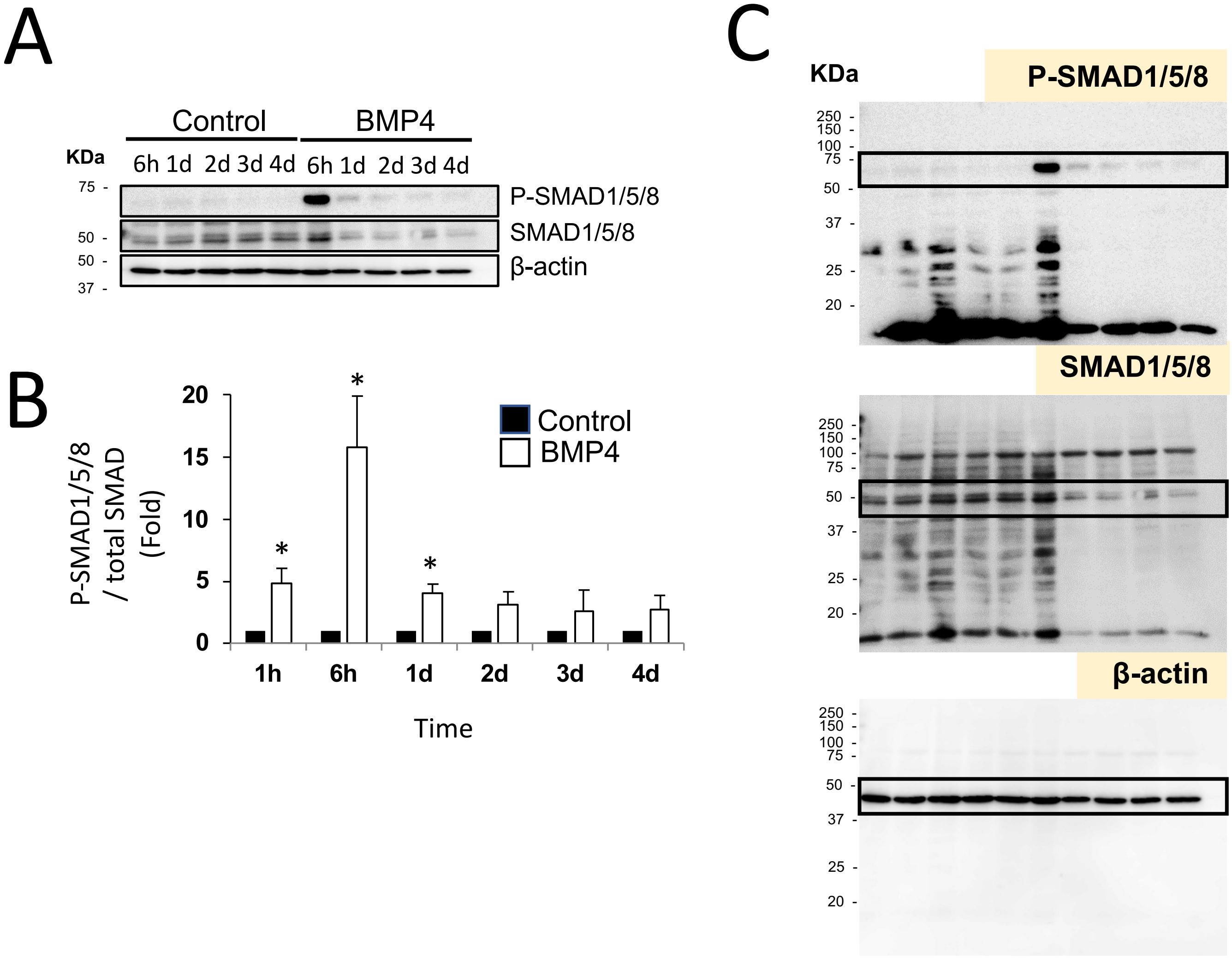

Supplementary Fig. S6. BMP4 transiently activates the P-SMAD canonical pathway in AH-NSPCs. (A) Representative Western immunoblot showing the transient phosphorylation of SMAD proteins. Whole cell lysates from AH-NSPCs treated with BMP4 were separated by SDS-PAGE and blotted sequentially with antibodies against P-SMAD1/5/8, total SMAD1/5/8 and β-actin as loading control. **(B)** P-SMAD1/5/8 levels normalized to total SMAD1/5/8 protein levels. Data are shown as the fold increase of the value for BMP4 treated cells vs. control cells at each time point (average ± sem, n=3). Values for the 1h time point were quantified from shorter treatments as shown in Supplementary Fig. S5 (average ± sem, n=3). **(C)** Original full-length Western immunoblots showing the cropped region used to compose the final image displayed in (A).

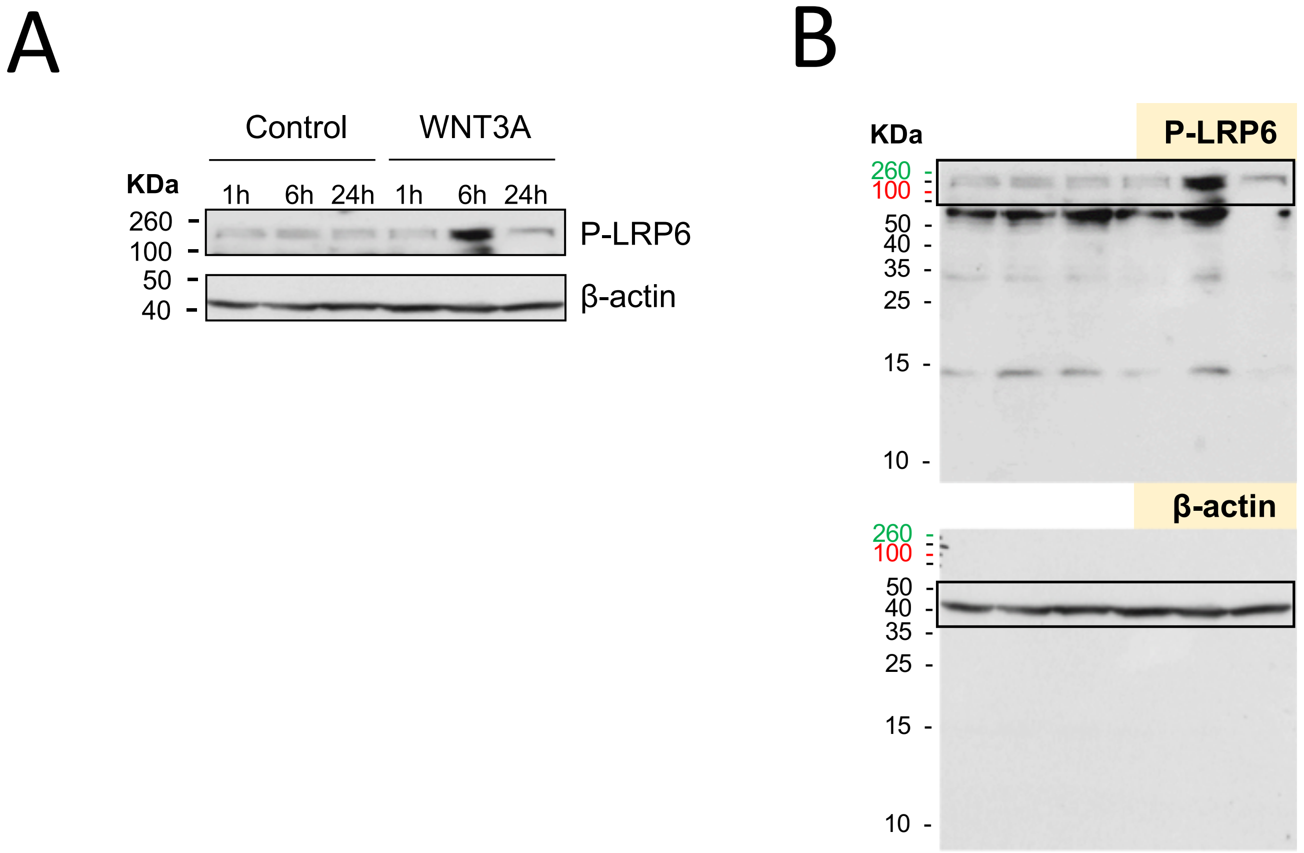

Supplementary Fig. S7. WNT3A activates the WNT canonical pathway in AH-NSPCs. (A) Western immunoblot shown in Figure 5D. Whole cell lysates from AH-NSPCs treated with WNT3A (100 ng/ml) for 1, 6 or 24 hours were separated by SDS-PAGE and blotted sequentially with antibodies against P-LRP6 and β-actin as loading control. **(B)** Original full-length Western immunoblots showing the cropped region used to compose the final image displayed in (A).

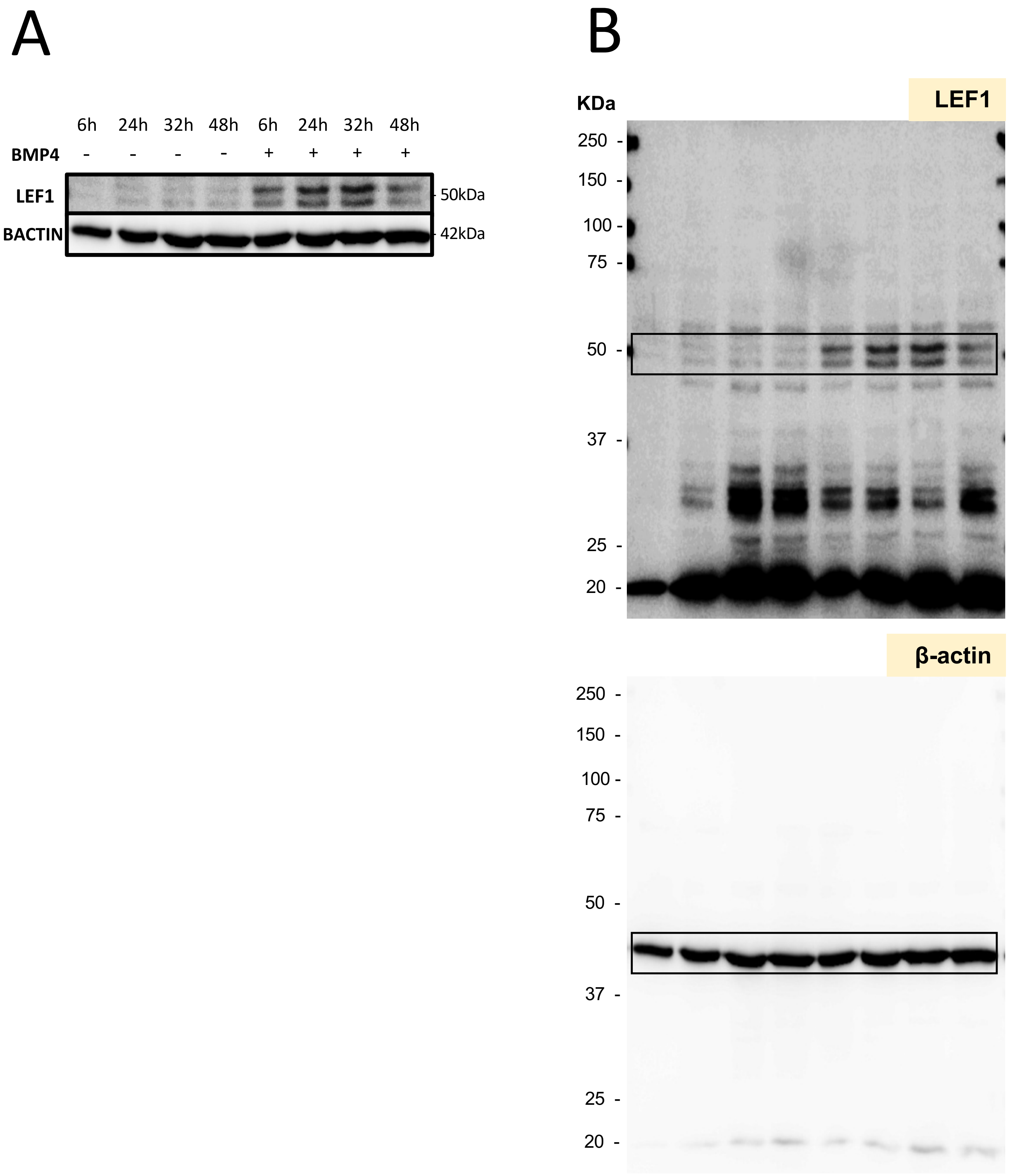

Supplementary Fig. S8. BMP4 treatment increases LEF1 protein levels. (A) Western immunoblot shown in Figure 7B. Whole cell lysates from AH-NSPCs treated with BMP4 at the indicated time points were separated by SDS-PAGE and blotted sequentially with antibodies against LEF1 and β-actin as loading control. **(B)** Original full-length Western immunoblots showing the cropped region used to compose the final image displayed in (A).

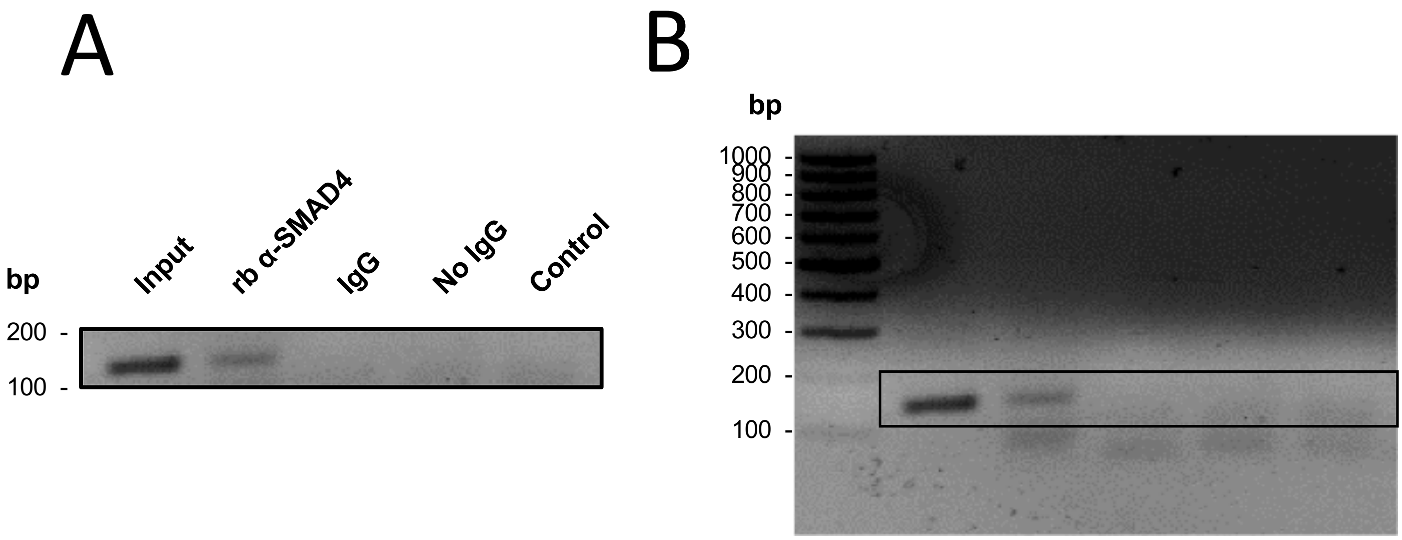

Supplementary Fig. S9. *Lef1* is a direct target of BMP4 canonical signalling in AH-NSPCs. (A) ChiP assay shown in Figure 7F. The ChIP assay was performed employing BMP4-treated AH-NSPCs using a SMAD4 rabbit antibody and rabbit IgG as a control (IgG)**.** Aditional controls were: Input chromatin, ChIP assay without antibody (No IgG) and PCR without template (Control). For the PCR amplification of the precipitated material, the underlined primers in Figure 7D were employed. **(B)** Original full-length agarose gel electrophoresis showing the cropped region used to compose the final image displayed in (A). Bp, base pairs.
